# Identification of novel transcripts with differential dorso-ventral expression in *Xenopus *gastrula using serial analysis of gene expression

**DOI:** 10.1186/gb-2009-10-2-r15

**Published:** 2009-02-11

**Authors:** Fernando Faunes, Natalia Sánchez, Javier Castellanos, Ismael A Vergara, Francisco Melo, Juan Larraín

**Affiliations:** 1 Center for Cell Regulation and Pathology and Center for Aging and Regeneration, Facultad de Ciencias Biológicas, Pontificia Universidad Católica de Chile, Alameda 340, Santiago, 8331150, Chile; 2 Laboratorio de Bioinformática Molecular, Depto. Genética Molecular y Microbiología, Facultad de Ciencias Biológicas, Pontificia Universidad Católica de Chile, Alameda 340, Santiago, 8331150, Chile

## Abstract

**Background:**

Recent evidence from global studies of gene expression indicates that transcriptomes are more complex than expected. *Xenopus *has been typically used as a model organism to study early embryonic development, particularly dorso-ventral patterning. In order to identify novel transcripts involved in dorso-ventral patterning, we compared dorsal and ventral transcriptomes of *Xenopus tropicalis *at the gastrula stage using serial analysis of gene expression (SAGE).

**Results:**

Of the experimental tags, 54.5% were confidently mapped to transcripts and 125 showed a significant difference in their frequency of occurrence between dorsal and ventral libraries. We selected 20 differentially expressed tags and assigned them to specific transcripts using bioinformatics and reverse SAGE. Five mapped to transcripts with known dorso-ventral expression and the frequency of appearance for these tags in each library is in agreement with the expression described by other methods. The other 15 tags mapped to transcripts with no previously described asymmetric expression along the dorso-ventral axis. The differential expression of ten of these novel transcripts was validated by *in situ *hybridization and/or RT-PCR. We can estimate that this SAGE experiment provides a list of at least 86 novel transcripts with differential expression along the dorso-ventral axis. Interestingly, the expression of some novel transcripts was independent of β-catenin.

**Conclusions:**

Our SAGE analysis provides a list of novel transcripts with differential expression in the dorso-ventral axis and a large number of orphan tags that can be used to identify novel transcripts and to improve the current annotation of the *X. tropicalis *genome.

## Abstract 2

Comparison of dorsal and ventral transcriptomes of Xenopus tropicalis gastrulae using serial analysis of gene expression provides at least 86 novel differentially expressed transcripts.

## Background

Embryonic dorso-ventral patterning has been extensively studied in *Xenopus laevis *[1]. Sperm entry produces a cortical rotation that establishes the future dorsal and ventral sides of the embryo through dorsal localization of maternal determinants such as β-catenin [2]. The activation of β-catenin signaling in the dorsal side and Nodal signaling in the equator of the embryo generates the Spemann organizer (dorsal blastopore lip). Spemann and Mangold demonstrated in 1924 that this region of the embryo is able to generate double axes when it is grafted to the ventral side [3,4].

Since the discovery of the organizer, several screens have been carried out to identify genes involved in dorso-ventral patterning [5-9]. All these screens were made without genome information and took advantage of very simple treatments that result in increased dorso-anterior or ventral development, such as LiCl incubation (increasing Wnt signaling) or UV irradiation, respectively [10,11]. A functional screen designed for the identification of dorsal-specific genes was performed by Harland and collaborators in the early 1990s [8]. Pools of cDNA prepared from LiCl-treated embryos were injected in UV-irradiated embryos. Pools able to rescue UV-treated embryos were analyzed by sib-selection until individual cDNAs were isolated. This approach allowed the identification of some dorsal genes, including *noggin *and *Xnr3 *[7,12].

Another approach, used by De Robertis's laboratory, was to perform differential screens. Duplicated filters from a dorsal lip cDNA library were hybridized with dorsalized or ventralized probes from LiCl- or UV-treated embryos, respectively. This screen identified the dorsal gene *chordin *[6]. Subsequently, other screens have been performed and, at present, several genes involved in dorso-ventral patterning are known, most of them being differentially expressed between the dorsal and ventral sides [3]. However, the fact that genes isolated in some screens were not isolated in others suggests that the identification of genes with dorsal and ventral asymmetric expression has not been exhausted.

Most of the previous screens have used LiCl-dorsalized embryos and recent evidence has shown that there are dorsal genes independent of the β-catenin pathway [13]. Therefore, additional signaling pathways contribute to organizer formation, including the Nodal and bone morphogenetic protein (BMP) signaling pathways [1]. In summary, previous screens, although successful, have been biased toward the detection of abundant, active or β-catenin-dependent genes. This indicates that our knowledge of the transcriptome involved in dorso-ventral patterning is not complete and that a global transcriptome analysis can contribute to increase the catalogue of genes implicated in this process.

More recently, several microarray and macroarray studies have been performed in *Xenopus *embryos with different experimental set-ups [14-22], including comparison between dorsal and ventral regions [13,14,16,23]. Many genes have been identified in these studies, confirming that global approaches can be successfully used to explore transcriptomes and to assist the discovery of new genes.

Another methodology for global analysis of transcriptomes is serial analysis of gene expression (SAGE). This sequencing-based technique generates 14-bp sequences (tags) to evaluate thousands of transcripts in a single assay [24]. One of the main advantages of SAGE, when compared to microarrays, is that it detects unknown transcripts, because it does not require prior knowledge of what is present in the sample under analysis. In addition, SAGE is a quantitative method. The frequency of tag occurrence observed in a SAGE library is a measure of the expression level of each transcript, allowing comparative analysis of two or more experimental conditions. SAGE has been used to study several biological processes in different model organisms [24-30]; however, no SAGE experiments have been performed in *Xenopus*.

One of the most difficult steps in SAGE is the process of tag-mapping, which consists of the unambiguous assignment of each experimental tag to a transcript [31,32]. Most of the published SAGE experiments have used software based on public transcript databases, such as SAGEmap [33], to perform the tag-mapping process. However, when using this approach, many experimental tags do not match to transcript databases [32] because our current knowledge of transcriptomes is only partial. To overcome this problem, the complete genome sequence can be used for tag-mapping [31,34,35]. This strategy favors the identification of novel transcripts, which in turn helps to improve the current annotation. At present, a draft of the *Xenopus tropicalis *genome is available [36] and it can be used to perform tag-mapping.

In order to have a more comprehensive knowledge of the transcriptome involved in dorso-ventral patterning, we performed a SAGE experiment with *X. tropicalis *embryos. Two libraries, from dorsal and ventral explants isolated from gastrula stage embryos, were prepared and a total of 63,222 experimental tags were obtained. The process of tag-mapping was performed using both the complete *X. tropicalis *genome sequence and available transcript databases. We found that 45.5% of experimental tags could not be mapped with confidence to transcript databases and probably represent novel transcripts. A comparison between SAGE libraries showed that 125 tags have a significant differential frequency of occurrence between the two libraries, 117 of which mapped to transcripts not previously linked to dorso-ventral patterning. Using bioinformatics or reverse SAGE (rSAGE), transcripts corresponding to 20 differentially expressed tags were identified. Five of them map to genes with known dorso-ventral expression and the frequency of appearance for these tags in each library is in agreement with the expression described by other methods. The other 15 tags map to novel transcripts. The differential expression of ten transcripts was validated by *in situ *hybridization and/or RT-PCR in *X. tropicalis *and *X. laevis*. From these analyses we can estimate that our SAGE experiment provides a list of at least 86 novel transcripts with differential expression in the dorso-ventral axis. Interestingly, the expression of three transcripts was independent of β-catenin signaling. To the best of our knowledge, this is the first SAGE experiment in *Xenopus *and novel transcripts identified in this study are potential candidates to have a role in dorso-ventral patterning.

## Results

### Analysis of SAGE libraries and tag-mapping

SAGE libraries were generated from total RNA of 500 dorsal and 500 ventral explants isolated from *X. tropicalis *embryos at stage 10. A total of 1,265 and 1,018 colonies from each library were sequenced, respectively (Table 1). The percentage of duplicated ditags and linker tags indicated that our libraries were properly prepared (Table 1). Duplicated ditags were considered once and linker tags were eliminated from the analysis. In total, 63,222 tags were obtained, corresponding to 23,766 different tag sequences (experimental tags).

**Table 1:** Description of dorsal and ventral SAGE libraries

SAGE library	Dorsal	Ventral	Total
Sequenced colonies	1,265	1,018	2,283
Repeated ditags	2,183 (12.2%)	359 (2.2%)	2,542 (7.4%)
Ditags*	15,773	16,057	31,830
Tags^†^	31,538	32,104	63,642
Linker tags	363	57	420
Total experimental tags	31,175	32,047	63,222
Unique experimental tags	14,546	14,486	23,766
Experimental tags matching to the genome	14,352	14,347	23,455

SAGE libraries were prepared from total RNA of dorsal and ventral explants of *X. tropicalis *gastrula. Concatemer sequences were processed for tag extraction and comparison between libraries. *Repeated ditags were considered only once. ^†^Tags including 'N' in the sequence were not considered (eight tags in the dorsal library and ten tags in the ventral library).

Most of the experimental tags were singletons (68.8%; tags with count equal to 1), as typically observed in SAGE experiments [32]. Singletons probably represent transcripts of low abundance. Recently, experimental estimation indicated that the error rate of sequencing in SAGE is approximately 1.67% per tag [37], indicating that low count tags are derived in most cases from real transcripts [38,39]. For this reason, singletons in our SAGE experiment were included for global analysis.

The process of tag-mapping, which consists of the assignment of each experimental tag to a transcript, is one of the most difficult steps in SAGE. The tag-mapping procedure was specifically designed to take advantage of the availability of a draft of the *X. tropicalis *genome sequence [36], its current annotation in Ensembl [40], and several transcript databases that included 28,657 sequences from Ensembl, 7,976 mRNA sequences from the National Center for Biotechnology Information (NCBI), 42,654 sequences from Unigene [41] and 41,921 full-length expressed sequence tag (EST) clusters from the Gurdon Institute [42]. A list of virtual tags for each database was prepared. The bioinformatics approach used here is similar to that previously published for tag-mapping in yeast [31], but with some modifications (see Materials and methods).

**Figure 1:**
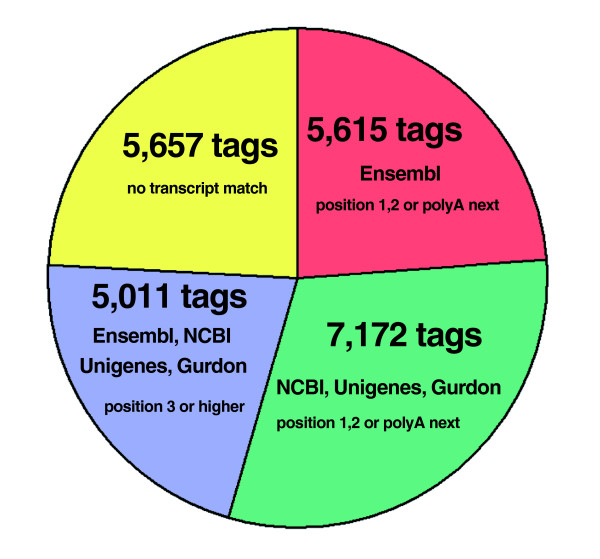
Tag-mapping of experimental tags to *X. tropicalis *genome and transcript databases. All different experimental tags (23,766 tags) were mapped first to the genome of *X. tropicalis *and those without a match (311 tags) were discarded from further analysis. The remaining experimental tags that presented one or more matches to the genome (23,455 tags; 100%) were then mapped to the Ensembl modified database, and only those tags found in the first or second positions from the 3'-end of the RNA sequence or belonging to the polyA-next category (see Materials and methods for details) were selected and reported as mapping to this transcript database (5,615 tags; 23.9%; red). The remaining tags that did not exhibit a match to the transcripts in the Ensembl modified database (17,840; 76.1%) were then searched with the same restraints mentioned above in the joint set composed of the NCBI (mRNAs), Unigene (clusters of mRNAs and ESTs) and Gurdon databases (clusters of ESTs). A total of 7,172 tags (30.6%) were found to match to positions 1, 2 or poly-A next in the transcripts from this set (green). The remaining tags without a match to these databases (10,668; 45.5%) were then re-mapped against the complete set of transcripts (a complete joint set of RNAs composed of Ensembl, NCBI, Unigene and Gurdon databases), but with the restraint that the mapping must occur to position 3 or above in a transcript. A total of 5,011 tags (21.4%) that fulfilled these conditions were obtained (blue). The remaining 5,657 (24.1%) tags mapped to the genome, but did not map to any known transcript (yellow).

The list of genomic virtual tags contained 892,958 different tag sequences. Of the experimental tags, 23,455 tags (98.7%) match to the genomic virtual tag database. The small set of tags (1.3%) that do not match to the genome could be explained by post-transcriptional processing (for example, splicing) or sequencing errors. For tag-mapping, the set of 23,455 experimental tags was used (Figure 1). Only 763 tags (3.3%) matched to a single genomic position and 11,893 tags (50.7%) had 15 or more genomic matches. This confirms that the accurate and unambiguous mapping of 14-nucleotide SAGE tags onto a genome sequence with a size of 1.7 Gb is a complex process.

The current Ensembl annotation was used to accomplish tag-mapping to known cDNAs and to determine the tag position from the 3'-end in the cDNA. Considering that in the SAGE protocol experimental tags should mainly derive from the 3'-most CATG position in each transcript, knowledge of the 3'-untranslated region (UTR) sequence in each transcript is essential to achieve accurate tag-mapping. Although the Ensembl annotation used here contains a large number of transcripts (28,657 cDNA sequences), only 14.2% (4,067 sequences) of them have a known 3'-UTR. As an attempt to circumvent this problem, we assigned the 3'-UTR for the remaining transcripts that lack this information based on the known 3'-UTRs available for *X. tropicalis *(see Materials and methods). Virtual tags were extracted from this modified Ensembl cDNA database, and the position for each tag relative to the 3'-end was recorded. When experimental tags were searched in this modified database, we found that only 23.9% of them (Figure 1, red; 5,615 tags) matched to positions 1 or 2 or immediately upstream of an internal polyA tract (defined as 'polyA-next'). We considered polyA-next tags because it has been demonstrated that reverse transcription can occur from these internal polyA stretches [43]. Tags matching to position 2 in a transcript were included, because tags from this position can be experimentally obtained at a low but still significant frequency [31].

In addition to Ensembl cDNAs, other transcript databases of *X. tropicalis *are also available, but not yet mapped to the genome by Ensembl. These transcripts were also used as a source for mapping the experimental tags. Experimental tags with no match to positions 1, 2 or polyA-next in the Ensembl modified database were mapped to mRNAs from NCBI, EST cluster sequences from Unigene and full-length ESTs from the Gurdon Institute. We found that 30.6% of experimental tags (Figure 1, green; 7,172 tags) matched to position 1, 2 or polyA-next in these transcripts. In summary, this analysis showed that only 54.5% of the experimental tags could be assigned with high confidence to known transcripts (Figure 1, red and green). In consequence, a confident mapping was not possible for 45.5% of the experimental tags (Figure 1, blue and yellow; 10,668 tags) and these were designated as orphan tags. This amount of orphan tags is similar to those observed in other SAGE experiments [32]. Although 21.4% of experimental tags (Figure 1, blue; 5,011 tags) could be found in transcript databases at higher positions (that is, 3 and above, but not polyA-next), these tags were probably not experimentally derived from those transcripts. This is based on the fact that tags derived from positions 3 or above are not experimentally observed in all SAGE libraries published in yeast [31]. This set of 10,668 orphan tags might represent unknown transcripts of low abundance, suggesting that the current annotation of *X. tropicalis *is far from complete.

### Distribution of experimental tags derived from known dorso-ventral genes

**Figure 2:**
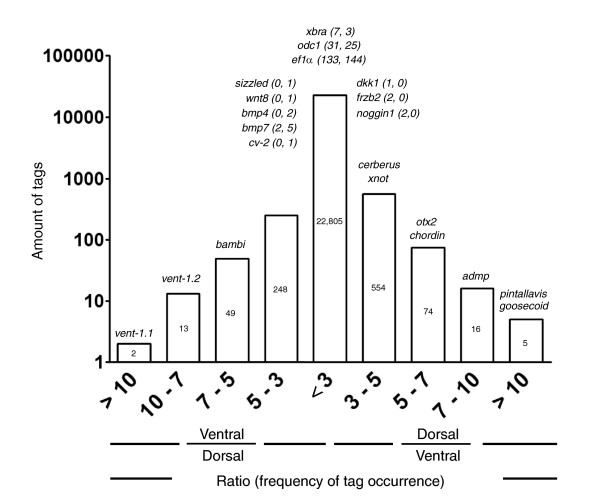
Comparison of the normalized frequencies of tag occurrence between dorsal and ventral SAGE libraries. Tag frequencies were normalized with respect to the total tags in each library (31,175 total dorsal tags and 32,047 total ventral tags), grouped according to their ratio of frequency of occurrence in both libraries and plotted against the counts of tags in each category. The number of tags is indicated inside each bar. Expected tags for known genes with a role in dorso-ventral patterning and control genes are indicated for each category. For these genes, the frequency of occurrence in each library is indicated in parentheses (tag frequency in dorsal library; tag frequency in ventral library).

Our main interest is to identify novel transcripts with differential expression in the dorso-ventral axis of *Xenopus *during early development. For this, we plotted a histogram for the normalized ratio of the frequency of occurrence of tags in the dorsal and ventral libraries (Figure 2). We found that 96% of the experimental tags (22,805 tags) have a ratio of frequency of occurrence between both libraries smaller than threefold. Only 961 tags have a ratio of threefold or larger between libraries. From these, 649 tags appeared more frequently in the dorsal library.

As a first step to validate the results of our SAGE experiment, sequences of some transcripts known to be differentially expressed along the dorso-ventral axis were analyzed and the potential tag from the 3'-most CATG position was extracted (Supplementary Table 1 in Additional data file 1). All possible genomic positions were analyzed for these tags and it was not possible to make a second transcript assignment for any of them (data not shown). Additionally, when possible, the 15th base of each tag was also considered to give more reliability to the tag assignment. Tagging enzymes can digest 14 or 15 bases downstream of the recognition site; thus, the 15th base can be used to decrease ambiguity in particular cases [35,44].

Remarkably, all tags extracted from known genes presented the expected distribution in the two SAGE libraries (Figure 2; Supplementary Table 1 in Additional data file 1). Tags derived from known dorsal genes, such as *pintallavis*, *goosecoid*, *admp*, *chordin*, *Otx2*, *cerberus *and *Xnot*, appeared more frequently in the dorsal library. Tags derived from known ventral genes, such as *vent-1.1*, *vent-1.2 *and *bambi*, appeared more frequently in the ventral library (Figure 2). Although tags derived from other known genes appeared with low frequency and had no statistically significant difference, their trend of appearance was correct (*dkk-1*, *frzb2*, *noggin *appeared more frequently in the dorsal library, and *sizzled*, *bmp4*, *bmp7*, *crossveinless-2 *and *Wnt8 *appeared more frequently in the ventral library). Furthermore, genes known to be expressed without difference in the dorso-ventral axis at the gastrula stage, such as *xbra*, *ef1a *and *odc1*, had similar frequencies of occurrence in dorsal and ventral libraries. These results indicate that our SAGE libraries were properly prepared.

### Identification of transcripts corresponding to experimental tags with differential frequency of occurrence between dorsal and ventral SAGE libraries

To identify novel transcripts that are expressed differentially between dorsal and ventral poles, we generated a list of tags having a statistically significant difference of occurrence in their dorsal and ventral libraries. We obtained 180 tags with a statistically significant difference (*p*-value < 0.05) by three independent tests [29,45,46](Additional data file 2). In order to increase the discovery rate of new genes with differential expression in dorsal and ventral poles, we removed from the list those tags with large counts but low fold-ratio between libraries (see Materials and methods). Though arbitrary, we applied this procedure to favor the characterization of novel transcripts previously not identified. After applying this filtering process, we ended up with a final list of 125 selected tags that were sorted according to their *p*-values and named *DV01*-*DV125 *(Supplementary Table 2 in Additional data file 1; Additional data file 2).

Bioinformatics tag-mapping showed that 105 of the 125 selected tags could be assigned confidently to known transcripts, even though most of them have several matches to the genome sequence (Supplementary Table 2 in Additional data file 1). A total of 18 tags were not confidently mapped to any known transcript and two tags were not found in the genome. Remarkably, among these 125 tags, only 8 tags mapped to genes with known function in dorso-ventral patterning (*pintallavis *(*DV01*), *vent-1.1 *(*DV03*), *goosecoid *(*DV06*), *admp *(*DV10*), *vent-1.2 *(*DV15*), *bambi *(*DV57*), *Otx2 *(*DV85*) and *zic3 *(*DV93*)).

Although many tags were confidently assigned to transcripts through bioinformatics approaches, we decided to experimentally confirm these predictions. For this we used rSAGE, a PCR-based method that allows the extension of a tag sequence towards the 3'-end of a transcript [47]. The rSAGE technique was performed for the first 18 of the 125 selected tags (Tables 2 and 3; Supplementary Table 3 in Additional data file 1), but it was successful in only 14 cases (Supplementary Table 3 in Additional data file 1), where the corresponding transcript was clearly identified (Table 3). The results obtained with rSAGE and our bioinformatics method for tag-mapping were concordant for 10 of the 11 tags for which there was information from both methods (*DV01*, *DV06*, *DV07*, *DV09*, *DV10*, *DV12*, *DV13*, *DV16*, *DV17 *and *DV18*). For two tags (*DV04 *and *DV14*), only rSAGE provided transcript information. For *DV08*, rSAGE allowed the selection of one out of two possible transcripts that were previously assigned through bioinformatics (Table 3). Only for *DV05 *rSAGE and bioinformatics were not concordant. Additionally, the use of the 15th base of each tag confirmed the tag assignments for almost all transcripts, with the exception of *DV04*. In summary, 17 out of 18 tags could be confidently mapped to their transcripts with one or both tag-mapping approaches (Table 3). No confident assignment for *DV02 *was possible.

**Table 2:** Set of selected tags and ratios between SAGE libraries

ID	Dorsal frequency	Ventral frequency	Normalized ratio*	*p*-value eSAGE^†^
*DV01*	34	2	17.5	6.65 e-9
*DV02*	20	1	20.6	8.53 e-6
*DV03*	0	15	-14.6	3.8 e-5
*DV04*	18	2	9.3	0.0001
*DV05*	20	3	6.9	0.0002
*DV06*	11	0	11.3	0.0004
*DV07*	9	0	9.3	0.0017
*DV08*	9	0	9.3	0.0017
*DV09*	11	1	11.3	0.0030
*DV10*	8	0	8.2	0.0035
*DV11*	8	0	8.2	0.0035
*DV12*	13	2	6.7	0.0036
*DV13*	1	11	-10.7	0.0039
*DV14*	0	8	-7.8	0.0044
*DV15*	0	8	-7.8	0.0044
*DV16*	10	1	10.3	0.0056
*DV17*	12	2	6.2	0.0064
*DV18*	12	2	6.2	0.0064
*DV22*	1	10	-9.7	0.0072
*DV25*	2	12	-5.8	0.0086
*DV38*	1	9	-8.8	0.0132

The first 18 tags of the list of tags with significant differential frequency of occurrence between libraries are shown (ordered by increasing *p*-value). Three additional ventral tags are also included (*DV22*, *DV25 *and *DV38*). *Normalized ratio is the ratio of relative dorsal and ventral frequencies, considering 31,175 total dorsal tags and 32,047 total ventral tags. Negative numbers indicate a higher ventral frequency. ^† ^*p*-value given by the eSAGE software for the differential expression of each tag between both SAGE libraries.

**Table 3:** Set of selected tags, tag-mapping and experimental validation

ID	Matches to genome	Bioinformatics mapping	rSAGE mapping	*X. laevis *homologue	Validation
*DV01*	23	*pintallavis*	*pintallavis*	*pintallavis*	Positive control
*DV02*	14	3 EST clusters	-	ND	ND
*DV03*	10	*vent1.1*	-	*vent-1.1*	Positive control
*DV04*	26	No transcript	Scaffold_19023: 2428-2444	Not found	*In situ*
*DV05*	1,482	6 transcripts	Cluster Str. 39849	Not found	PCR and *in situ*
*DV06*	2	*goosecoid*	*goosecoid*	*goosecoid*	Positive control
*DV07*	1	*zcsl-2*	*zcsl-2*	*LOC496356*	False positive
*DV08*	82	*LOC496648/ubadc1*	*ubadc1*	*MGC115132*	False positive
*DV09*	20	*sox11*	*sox11*	*sox11*	*In situ*
*DV10*	7	*admp*	*admp*	*admp*	Positive control
*DV11*	3	*LOC100124861*	-	*MGC82245*	False positive
*DV12*	9	*LOC549498*	*LOC549498*	*MGC115377*	*In situ*
*DV13*	12	*Id2*	*id2*	*id2*	PCR and *in situ*
*DV14*	10	No transcript	Cluster Str.3968	*MGC82755*	False positive
*DV15*	11	*vent1.2*	-	*vent1.2*	Positive control
*DV16*	3	*MGC147163*	*MGC147163*	*MGC81848*	PCR and *in situ*
*DV17*	11	*Str.45862/Str.40022*	*Str.45862/Str.40022*	Not found	PCR
*DV18*	68	*LOC548724*	*LOC548724*	*MGC116509*	*In situ*
*DV22*	9	*smagp*	ND	*mitotic phosphoprotein 22*	PCR and *in situ*
*DV25*	27	*cyclin A2*	ND	*MGC130969*	False positive
*DV38*	25	*nap1l1*	ND	*nap1*	PCR and *in situ*

Summary of the tag-mapping and experimental validation of selected tags. Dashes (-) indicate rSAGE failed to provide a longer and specific sequence. ND, not determined.

### Validation of dorso-ventral expression of novel transcripts identified by SAGE

Validation of the dorso-ventral differences observed by SAGE was carried out for 15 selected tags from Tables 2 and 3 using both semi-quantitative RT-PCR and *in situ *hybridization. We first selected 12 tags with confident assignment to transcripts not previously described to have asymmetric dorso-ventral expression (*DV04*, *DV05*, *DV07*, *DV08*, *DV09*, *DV11*, *DV12*, *DV13*, *DV14*, *DV16*, *DV17 *and *DV18*). Because most of these transcripts correspond to tags that are more abundant in the dorsal library, we decided to also include in the validation three additional tags that were more abundant in the ventral library and had a confident bioinformatics assignment (*DV22*, *DV25 *and *DV38*). It is worth mentioning that for 12 of these 15 selected transcripts, homologues in *X. laevis *were identified (*DV07*, *DV08*, *DV09*, *DV11*, *DV12*, *DV13*, *DV14*, *DV16*, *DV18*, *DV22*, *DV25 *and *DV38*) and that differential dorso-ventral expression at the gastrula stage has not been studied for any of these 15 transcripts in *Xenopus*. The expression of *DV09 *(*sox11*) and *DV13 *(*id2*) has been previously studied in *X. laevis*, but at the neurula and later stages [48,49]. For *DV38 *(*nap1*), its late expression pattern and role in haematopoiesis have been described in *X. laevis *[50,51]. Because this available information for *DV09*, *DV13 *and *DV38 *is useful for comparing with our results, we decided to include these transcripts in the selected set for validation of our SAGE data.

**Figure 3:**
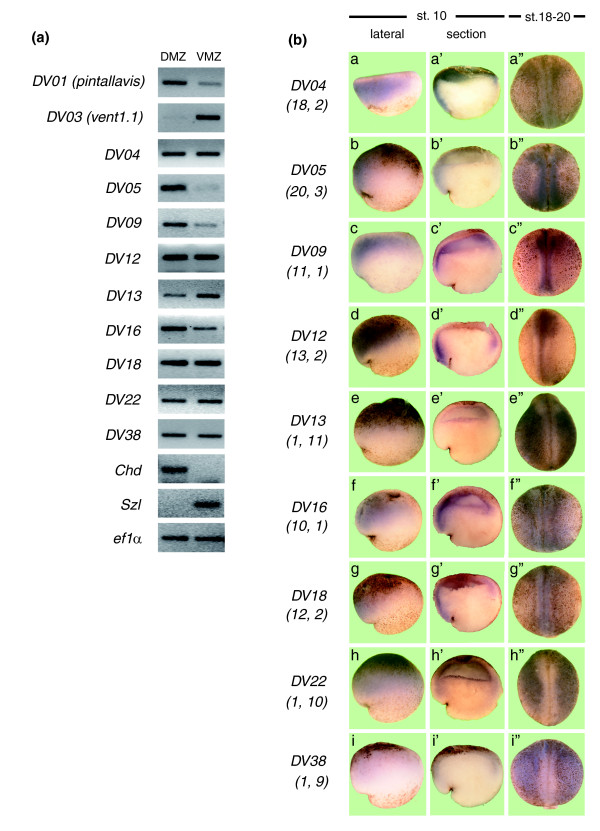
Verification of the differential expression of *X. tropicalis *transcripts identified by SAGE. **(a) **Total RNA was obtained from dorsal (DMZ) and ventral (VMZ) explants isolated from gastrula stage *X. tropicalis*. RT-PCR was performed using specific primers for each transcript. *DV01 *(*pintallavis*), *DV03 *(*vent-1.1*), *chordin *and *sizzled *were included as controls. **(b) ***X. tropicalis *embryos at stage 10 (a-i, a'-i'), and stages 18-20 (a"-i") were processed for *in situ *hybridization with specific probes for each transcript. (a'-i') Hemi-sections from embryos at the gastrula stage. (a-i, a'-i') Dorsal to the left and (a"-i") anterior is up. The frequency of occurrence in each library is indicated in parentheses below the name for each transcript (tag frequency in dorsal library; tag frequency in ventral library).

**Figure 4:**
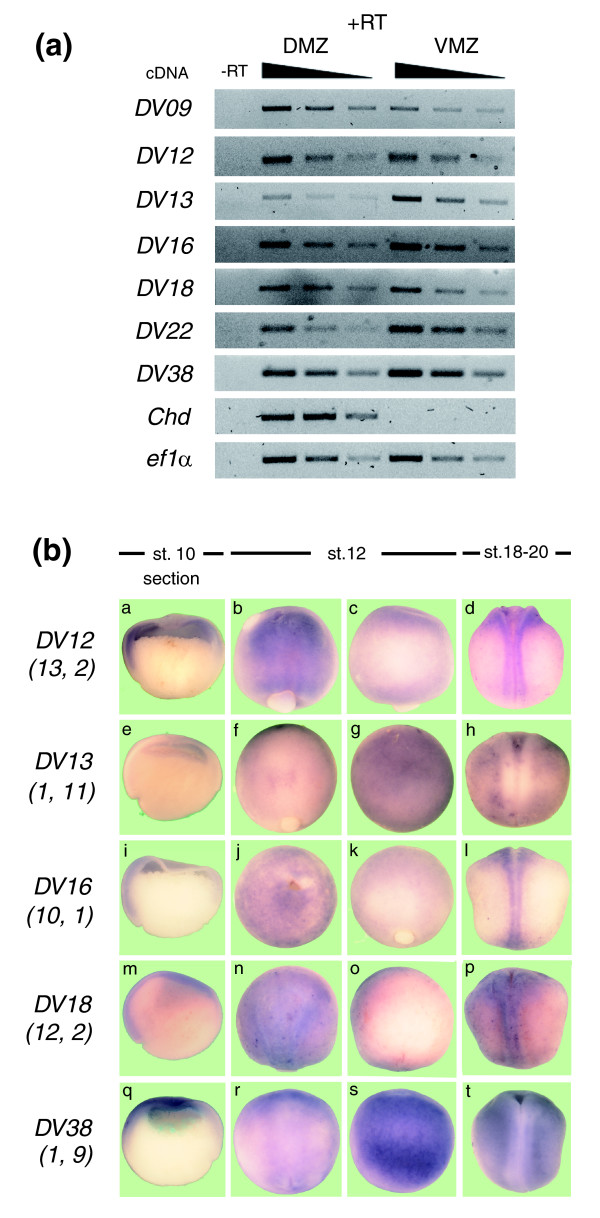
Verification of the differential expression of *X. laevis *homologues. **(a) **Total RNA was isolated from dorsal (DMZ) and ventral (VMZ) explants at the gastrula stage. RT-PCR was performed using specific primers for each transcript and different cDNA concentrations (serial dilutions of cDNA, 1:1, 1:2 and 1:4). *Chordin *was included as control. Reverse transcription in the absence (-RT) or presence (+RT) of reverse transcriptase for specificity of cDNA amplification. **(b) ***X. laevis *embryos at stage (st.) 10 (a, e, i, m, q; hemi-sections, dorsal to the left), stage 12 (b, c, f, g, j, k, n, o, r, s; anterior is up) and stages 18-20 (d, h, l, p, t; anterior is up) were processed for *in situ *hybridization with specific probes for each transcript. Stage 12 embryos are pictured from both sides relative to the blastopore to illustrate its asymmetric expression. Numbers under each transcript correspond to the frequency of occurrence in each SAGE library (tag frequency in dorsal library; tag frequency in ventral library).

As a first validation approach, we performed semi-quantitative RT-PCR analysis in dorsal and ventral explants from *X. tropicalis *and *X. laevis*. RT-PCR of *X. tropicalis *gastrula showed a clear difference for the transcripts derived from tags *DV05*, *DV09*, *DV13*, *DV16 *and *DV17 *(Figure 3a; Additional data file 3), confirming the SAGE results. Differential expression for *DV09*, *DV13*, *DV22 *and *DV38 *homologues was observed in *X. laevis *(Figure 4a). This partial validation of differential expression for some transcripts suggests that semi-quantitative RT-PCR may only be successful at identifying large differences in expression. Because of these results, and although more laborious, we decided to also use *in situ *hybridization in *X. tropicalis *and *X. laevis *as an alternative and complementary technique to experimentally validate the differences in gene expression observed by SAGE for some of the selected cases.

*In situ *hybridization analysis in *X. tropicalis *showed preferential dorsal expression at the gastrula stage for *DV04*, *DV05*, *DV09*, *DV12*, *DV16 *and *DV18 *(Figure 3b, panels a, b, c, d, f and g), in agreement with their higher frequency of occurrence in dorsal SAGE libraries (Table 2). Hemi-sectioned gastrulae embryos showed that these transcripts were preferentially expressed in the prospective neuroectoderm (Figure 3b, panels a', b', c', d', f' and g'). At later stages, all these transcripts were expressed in dorsal structures (Figure 3b, panels a", b", c", d", f" and g"). A similar expression pattern for *DV12*, *DV16 *and *DV18 *was observed in *X. laevis *at the gastrula stage (Figure 4b, panels a, i and m). Moreover, in *X. laevis *embryos at stage 12, differential expression along the dorso-ventral axis (perpendicular to the blastopore) was observed (compare panels b with c, j with k, and n with o in Figure 4b). Based on their early (Figure 4b, panels a, i and m) and late expression patterns (Figure 4b, panels d, l and p) showing exclusive localization to dorsal structures, we conclude that the expression observed at stage 12 is mainly in the dorsal side (that is, neural plate).

We also studied the expression of those tags that appear more frequently in the ventral libraries (*DV13*, *DV22 *and *DV38*). Using *in situ *hybridization, we did not detect differential expression for *DV13*, *DV22 *or *DV38 *at the gastrula stage in *X. tropicalis *(Figure 3b, panels e, e', h, h', i and i') and *X. laevis *(Figure 4b, panels e and q). However, at stages 18-20, these transcripts were excluded from dorsal structures both in *X. tropicalis *(Figure 3b, panels e", h" and i") and *X. laevis *(Figure 4b, panels h and t). Furthermore, *DV13 *and *DV38 *were already expressed asymmetrically at stage 12 in *X. laevis *(compare panels f with g and r with s in Figure 4b). *DV13 *and *DV38 *were also expressed ventrally at later stages (Figure 4b, panels h and t), suggesting that their expression at stage 12 is in the ventral side. Although ventral expression at stage 10 was not detected by *in situ *hybridization, RT-PCR analysis showed that ventral explants from *X. laevis *expressed higher levels of *DV13*, *DV22 *and *DV38 *(Figure 4a). The results obtained by *in situ *hybridization at later stages and RT-PCR analysis at the gastrula stage suggest that *DV13*, *DV22 *and *DV38 *correspond to ventral genes, thus validating the results observed by SAGE. In summary, we have experimentally demonstrated the differential expression of 10 of the 15 transcripts selected for validation. The expression of *DV07*, *DV08*, *DV11*, *DV14 *and *DV25 *was also evaluated by RT-PCR and/or *in situ *hybridization. We found that their distributions were not correlated with the frequency of occurrence observed for the original tag in the SAGE experiment (they were either expressed uniformly or with the opposite trend to the SAGE data). These five tags could correspond to false positives or incorrect tag-mapping.

In order to have an estimation of the false discovery rate of our SAGE experiment, we selected 20 tags with differential frequency of appearance between SAGE libraries and a confident assignment to specific transcripts. Five of them map to transcripts with known dorso-ventral expression (*pintallavis*, *vent1.1*, *goosecoid*, *admp *and *vent1.2*) and the frequency of appearance for these tags in each library is in agreement with the previously described expression. For that reason these tags were considered as true positives. The other 15 tags map to transcripts with no asymmetric expression along the dorso-ventral axes previously described. We have demonstrated experimentally (*in situ *hybridization and/or RT-PCR) that ten of these novel transcripts (*DV04*, *DV05*, *DV09*, *DV12*, *DV13*, *DV16*, *DV17*, *DV18*, *DV22 *and *DV38*) are differentially expressed along the dorso-ventral axis as predicted by our SAGE analysis. These ten tags/transcripts were also considered true positives. Only the expression of five of the transcripts experimentally studied (*DV07*, *DV08*, *DV10 DV14*, and *DV25*) did not correspond to the frequency of appearance between the SAGE libraries and, for this reason, are considered false positives. These results indicate that the false discovery rate is 25% (5 false positives out of 20 transcripts experimentally analyzed). Therefore, we can estimate that, from the set of 125 tags that have a significant difference of appearance in dorsal and ventral libraries, 31 tags could correspond to false positives and 94 tags could correspond to transcripts with differential dorso-ventral expression at the gastrula stage. Importantly, 86 tags of those expressed differentially correspond to novel transcripts.

### Regulation of expression by β-catenin of novel transcripts identified by SAGE

Many of the genes involved in dorso-ventral patterning were identified in previous screens that have used embryos dorsalized through activation of Wnt/β-catenin signaling. It has been proposed that β-catenin is the earliest signal in the formation of the organizer. However, other signaling pathways, such as Nodal (and inhibition of BMP signaling), are also involved in formation of the organizer [1,13].

**Figure 5:**
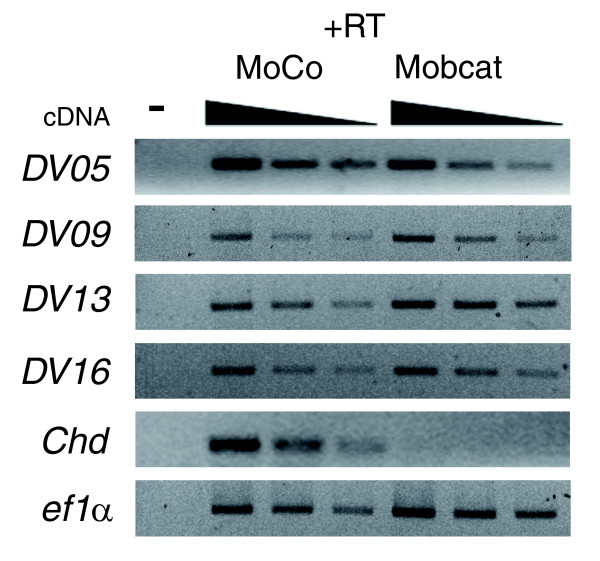
Effect of Wnt signaling on expression of novel transcripts. *X. tropicalis *embryos were injected at the two-cell stage with control and β-catenin morpholinos and total RNA was isolated at the gastrula stage. RT-PCR was performed by using specific primers for selected transcripts (serial dilutions of cDNA, 1:1, 1:2 and 1:4). Only transcripts for which a dorso-ventral expression difference was detected by RT-PCR were analyzed. *Chordin *was included as a positive control of a gene dependent on β-catenin. PCR in the absence (-) or presence of cDNA (+RT) from embryos injected with control (MoCo) and β-catenin (Moßcat) morpholinos.

To determine if the expression at the gastrula stage of some of the transcripts identified in this screen was β-catenin dependent, morpholinos against β-catenin mRNA were used [52,53]. *X. tropicalis *embryos were injected at the two-cell stage and cultured up to the gastrula stage. We performed RT-PCR analysis to compare the expression of transcripts in control and β-catenin morpholino-injected embryos. We studied transcripts whose differential expression was detected by RT-PCR between the dorsal and ventral sides (detection of a dorso-ventral difference indicates that RT-PCR conditions are sufficient to detect differences in gene expression; Figure 3a). Interestingly, the expression at the gastrula stage of the dorsal transcripts *DV05*, *DV09 *and *DV16 *were independent of β-catenin (Figure 5). Contrary to this, the ventral transcript *DV13 *was regulated by β-catenin signaling (Figure 5). These results indicate that the dorso-ventral expression of these novel transcripts is β-catenin independent.

## Discussion

### Analysis of SAGE data

Dorso-ventral patterning has been extensively studied in *Xenopus *embryos. Several screens have been performed to identify genes involved in this process. These screens, although successful, have probably detected the most abundant, active or Wnt-dependent genes; therefore, they do not provide complete knowledge of the transcript catalogue involved in dorso-ventral patterning.

More recently, global approaches such as microarray analysis have been used in *Xenopus *to study different biological processes and many genes have been identified [14-23]. Macroarray analysis suggested that novel pathways, additional to Wnt/β-catenin signaling, are involved in formation of the organizer [13]. The general conclusion of global studies of gene expression in all species is that transcriptomes are more complex than initially expected. One method of global analysis that can be used for studying gene expression is SAGE, and this methodology has never been used before in *Xenopus*. In contrast to microarrays, SAGE does not need previous information on transcriptomes; therefore, novel transcripts can be identified. Both methodologies, microarrays and SAGE, can be considered as complementary in successfully exploring the transcriptome.

We performed a SAGE experiment comparing libraries generated from dorsal and ventral explants of *Xenopus *gastrula. We used *X. tropicalis *due to the recent availability of its genome sequence, which allows a more accurate tag-mapping process, thus favoring the identification of novel transcripts. Our aim was to carry out a SAGE experiment as a proof of concept that several novel transcripts with differential expression along the dorso-ventral axis can be identified with this technique and that our knowledge of the genes involved in this process is far from complete.

This SAGE experiment cannot be considered a comprehensive study of gene expression during the early development of *X. tropicalis *because of the low sequencing level of our libraries compared to other SAGE experiments. Nevertheless, we believe that the comparison of these libraries gives us useful and reliable information. First, all tags derived from known genes with differential expression at this stage presented the correct distribution between both libraries, indicating that our SAGE libraries were properly prepared (Figure 2). The low sequence coverage of the experiment probably explains why not all tags derived from known genes with differential expression presented a significant difference in tag occurrence in both libraries. Second, we validated the SAGE data by RT-PCR and/or *in situ *hybridization for 10 of the 15 transcripts studied (Figures 3 and 4; Additional data file 3). We could not confirm the differential expression predicted by SAGE for five transcripts, either because they probably correspond to false positives or their tag-mapping was incorrect (for example, more than one transcript could produce the same tag). This suggests that although not all transcripts were confirmed (false positive rate of 25%), we have a reliable list of novel transcripts with differential dorso-ventral expression in *Xenopus *at the gastrula stage.

Comparison of our data to similar micro- and macroarray experiments [13-15,20,23] indicate that the pool of transcripts identified by both methods are different, giving support to the idea that these methodologies should be complementary to each other to acquire a complete knowledge of the transcriptome [32]. In addition, it seems that SAGE analysis is particularly efficient for the identification of novel transcripts. Microarray analysis of genes involved in neural induction (that is, dorsal genes) allowed the identification of 14 novel transcripts out of 32 that were validated [20]. In the case of the SAGE experiment presented here, 105 of the 125 tags represented differentially in both libraries mapped with high confidence to novel transcripts (Supplementary Table 2 in Additional data file 1).

It is not clear yet if the copy number of each SAGE tag accurately reflects the absolute quantity of the transcripts present in each sample [32]. Bias can be introduced by PCR amplifications, cloning and colony propagations. Nevertheless, we found that SAGE could detect differential expression of transcripts between dorsal and ventral explants. At the gastrula stage, *in situ *hybridization was able to detect dorsal localization of six transcripts (three of them also studied in *X. laevis*) that correspond to tags that appear more frequently in the dorsal library (*DV04*, *DV05*, *DV09*, *DV12*, *DV16 *and *DV18*), validating the SAGE data. When expression levels were analyzed by RT-PCR, higher dorsal expression only for *DV05*, *DV09*, *DV16 *and *DV17 *was demonstrated and no difference was observed for *DV04*, *DV12 *and *DV18*. Considering that our *in situ *analysis validated the dorsal expression of *DV04*, *DV12 *and *DV18*, an apparent contradiction between our RT-PCR analysis and SAGE is observed. However, our semi-quantitative RT-PCR analysis does not represent an exhaustive quantitative analysis and, in order to do more accurate comparisons, real time PCR should be used. In addition, although both RT-PCR and SAGE are PCR-based techniques, there are many differences in both protocols that preclude a perfect correlation from both methods. For instance, in the SAGE protocol, the PCR amplification is performed using primers that hybridize to the adaptors introduced into the cDNA instead of primers that are specific for internal sequences of each RNA as in the case of RT-PCR. More importantly, quantification in RT-PCR is indirect (EtBr staining) and for SAGE the frequency of appearance represents an analogue quantification of RNA amounts.

Regarding those tags that appeared more frequently in the ventral library, *in situ *hybridization did not detect dorso-ventral differential expression at the gastrula stage for any of them (*DV13*, *DV22 *and *DV38*). Interestingly, in *X. laevis *embryos asymmetric expression along the dorso-ventral axis was observed at stage 12. At stages 18-20 in both *X. tropicalis *and *X. laevis*, expression was clearly absent from neural tissues in the dorsal side and more enriched in ventral structures. Furthermore, RT-PCR analysis confirmed ventral expression of *DV13 *and *DV22 *at the gastrula stage. The fact that no difference at this stage was detected by *in situ *hybridization could be explained by the low levels of expression of these genes at this stage. Previous work has demonstrated that *DV13 *(*id3*) is regulated by BMP [48,54,55], a signaling pathway activated in the ventral side. Loss-of-function studies showed that *DV38 *has a role in hematopoiesis [51], which is consistent with its expression in ventral mesoderm. From all these observations, we conclude that the ventral enrichment of *DV13*, *DV22 *and *DV38 *predicted by our SAGE study was validated.

### Novel transcripts identified in this screen

The set of transcripts with verified differential expression in this study includes seven dorsal and three ventral transcripts of *X. tropicalis*. Some of them were also validated in *X. laevis*. A brief description of each of the validated tags/transcripts follows.

*DV04 *maps to a genomic region without an annotated gene. At this point no confident mapping has been possible to achieve. Therefore, further experimental examination is required to determine the transcript that is the origin of tag *DV04*.

*DV05 *corresponds to a putative transposase. A deep analysis of this sequence indicates that it corresponds to a Tc1-like transposable element and, interestingly, it is present 125 times in the genomic sequence (considering at least 95% of its sequence length and more than 97% sequence identity). We are at present trying to identify the complete sequence of this transcript and determining whether this tag is derived from a single or from several genomic positions. We also plan to perform some functional studies with this candidate gene. We found a highly similar sequence in zebrafish databases, but not in *X. laevis*.

*DV09 *corresponds to *sox11*, a transcript whose expression at the gastrula stage has not been previously described. *Sox11 *is dorsally expressed at neurula stages and has a role in neural induction [49], which is consistent with our data.

*DV12 *corresponds to a hypothetical protein (LOC549498), which has a domain with unknown function and has homologues in human (HSPC038 protein, 87% sequence identity) and mice (zinc finger protein 706, 87% sequence identity).

*DV13 *corresponds to *id2*, an inhibitor of differentiation protein 2. This protein has been described as a target of BMP signaling and its expression in *X. laevis *at later stages has already been reported [48,54,55]. Its dependence on BMP signaling is consistent with the higher ventral expression observed for this gene in the SAGE data.

*DV16 *corresponds to a hypothetical protein (LOC779989) that shares 80% and 77% sequence identity with thioredoxin reductase 1 of zebrafish and human, respectively, and 77% sequence identity with thioredoxin reductase 3 in mice. The role of thioredoxin reductases in embryogenesis and brain development has been described in mice [56,57].

*DV17 *corresponds to a transcribed locus without homologous transcripts described in other species.

*DV18 *corresponds to the hypothetical protein LOC548724, a putative membrane protein without any function described that shares 95% sequence identity with a membrane protein in zebrafish, mice and human.

*DV22 *corresponds to a small transmembrane and glycosylated protein homolog (encoded by *smagp*) without any role described in development and no clear sequence identity to other vertebrate proteins present in the NCBI databases.

*DV38 *corresponds to the nucleosomal assembly protein 1 like 1 (*nap1l1*) with 92% and 91% sequence identity to homologues in mice and human, respectively. Its expression pattern in *X. laevis *has been described [50]. Loss of function studies indicated that this transcript has a role in hematopoiesis [51], giving further support to our finding of *DV38 *as a ventral gene.

If we consider that differential expression for ten novel transcripts was verified by RT-PCR and/or *in situ *hybridization, it is plausible to propose that 86 of the tags with significant differences in frequency of occurrence between dorsal and ventral libraries would be derived from transcripts that have a real differential expression in these tissues. In addition, not all the tags from genes known to be expressed differentially along the dorso-ventral axis presented significant differential frequencies of occurrence in the SAGE libraries (*bmp4*, *bmp7*, *cerberus*, *sizzled*). From this, we can conclude that additional novel transcripts with differential expression between dorsal and ventral sides at the gastrula stage would be present in the set of tags that have a low count fold ratio. All together, this analysis suggests that the 86 tags that we considered in this study to be the group of potential novel transcripts involved in dorso-ventral patterning is probably still an underestimation of the set of genes involved in this process.

These results indicate that although dorso-ventral patterning has been extensively studied, novel transcripts with differential expression along the dorso-ventral axis in *Xenopus *could still be found by using global and unbiased studies of the transcriptome such as those performed with the SAGE technique.

### Regulation of these transcripts by Wnt signaling

Early Wnt signaling plays an essential role in establishing dorso-ventral patterning in *Xenopus *embryos [1]. Activation of this signaling pathway in the whole embryo produces dorsalization and its inhibition generates ventralized embryos. We found that the expression of three novel transcripts identified in this work was unaffected in morpholino β-catenin-ventralized embryos. Similar results were obtained by macroarray analysis, indicating that novel signaling pathways contribute to formation of the organizer [13]. It would be interesting to know if other pathways, such as the BMP and Nodal pathways, regulate the expression of these specific transcripts. In addition, other signaling pathways, such as the epidermal growth factor and fibroblast growth factor pathways, play a role in dorso-ventral patterning in other species [58,59]. Although the dorso-ventral differential expression suggests that these genes may have a function in this process, future functional studies will be necessary to address the potential role of these genes in dorso-ventral patterning.

### Tag-mapping to the genome and tags with no match to transcript databases

The availability of the *X. tropicalis *genome sequence supports the use of genomic approaches in *Xenopus*. In the case of SAGE, the genome sequence can be used to assist the tag-mapping process [31,34,35] and tags with no match to the transcript databases can be mapped to genomic positions, allowing the identification of novel genes. However, 14-nucleotide tags have multiple occurrences in the genome, making their assignment a difficult task. We calculated that 50.7% of experimental tags had 15 or more matches to the genome. Transcript databases were also used and experimental tags matching to transcripts in positions 1, 2 or polyA-next were confidently mapped to those transcripts and not to the other multiple genomic matches [31].

We found that 30.6% of experimental tags matching to the genome had no reliable match to Ensembl cDNAs (CATG position higher than 2). However, these tags matched to positions 1, 2 or polyA-next in mRNAs, clusters from NCBI or full-length sequence clusters from the Gurdon Institute. These results indicate that Ensembl annotation is incomplete, our 3'-UTR assignment is incorrect, or that unknown single nucleotide polymorphisms or splicing variants are present. Although our list contains only virtual tags from the genome and Ensembl cDNAs with their genomic location, we also have information from all other transcript databases, and they can be used to determine the assignments of particular tags. For example, the transcript *DV02*, one of the tags with large dorso-ventral differences, could not be mapped to Ensembl cDNAs, but it was found in 14 genomic positions and 3 EST clusters. Considering that rSAGE was not successful for this tag, these 14 genomic positions could be used to design specific primers and to experimentally determine which genomic position was the origin of the tag. Similar approaches can be performed for interesting tags with a low number of occurrences in the genome, showing the usefulness of using the genome sequence.

Remarkably, 45.5% of the experimental tags we obtained have no reliable match to any transcript. This value is similar to the ones obtained in other SAGE experiments [32]. Of the tags with no match to the transcript databases, 86.5% (4,893 of 5,657) are singletons. Whether these tags derive from true novel transcripts of low abundance or from sequencing errors is at present still under debate. It has been demonstrated that experimental errors in SAGE are low (1.67%) and most tags derive from true transcripts [37-39]. Even if a fraction of these correspond to SAGE errors, many of these tags could be derived from true novel transcripts, splicing events or editing, showing that our knowledge of the transcriptome is not complete. Experimental approaches such as rSAGE can probably be used to perform tag-mapping of these orphan tags. When we used rSAGE to confirm *in silico *assignments, we obtained information for 14 of 18 tags, an efficiency that is similar to that described by the inventors of this technique (66%; 131 of 200 orphan tags) [47]. The experimental assignment of these tags to specific genomic positions may not be useful only to identify novel transcripts but also to better estimate the 3'-end of annotated transcripts without a known 3'-UTR, thus improving the *X. tropicalis *genome annotation.

## Conclusion

This study provides a list of novel transcripts with differential expression in the dorso-ventral axis of *Xenopus *at the gastrula stage, some of which are β-catenin independent. These transcripts constitute interesting candidates for further functional studies. Also, the set of tags with no match to the transcript databases can be used to identify novel genes expressed at the gastrula stage and to improve the current genome annotation of *X. tropicalis*.

## Materials and methods

### Embryo manipulations

Natural and *in vitro *fertilizations of *X. tropicalis *were performed as described [52,60]. *In vitro *fertilizations, embryo culture, microinjections, explant culture and *in situ *hybridizations of *X. laevis *were performed as described [61]. Probes for *in situ *hybridizations were synthesized from PCR products cloned by using specific primers or from clones in the NIBB *Xenopus *database [62] (Supplementary Table 4 in Additional data file 1). Antisense morpholino oligonucleotides targeted to β-catenin were used as described [52,53].

### Preparation of SAGE libraries and SAGE data processing

Total RNA from 500 dorsal and 500 ventral explants from *X. tropicalis *gastrula (stage 10+) was isolated using Trizol (Invitrogen, Carlsbad, CA, USA). Correct purification of dorsal and ventral RNA was checked by RT-PCR of *chordin*, *sizzled *and *ef1α*. Total RNA (40 μg) of each sample was used for the SAGE protocol. SAGE libraries were prepared essentially as described [24] by using the I-SAGE kit (Invitrogen, Carlsbad, CA, USA) according to the manufacturer's instructions. Restriction enzymes *Nla*III and *Bsm*FI were used for tag generation. Concatemers were cloned into pZerO-1 and sequenced on a ABI PRISM 3700/3730xl system (Agencourt Bioscience Inc, Beverly, MA, USA). Concatemer sequences with Phred quality values larger than 20 were processed separately by eSAGE software [63] and SAGE2000 v4.5 software to extract the tags and to remove duplicated ditags and linker tags. The statistical significance of the differential frequency of occurrence was assessed using three different statistical tests [29,45,46]. The final set of unique experimental tags consisted of 23,766 sequences of 14 nucleotides each. Considering that the number of total tags sequenced is similar in both libraries (31,175 total dorsal tags and 32,047 total ventral tags), we indicate the absolute observed frequency of occurrence in all tables and figures. However, for statistical tests and fold-ratio analysis, the normalized ratio is considered (Table 2). Tag frequency equal to zero in a library is considered equal to 1 for normalization. The list of tag sequences, along with their observed frequency in each library, is included in Additional data file 4. The comparison between extraction of 14-nucleotide and 15-nucleotide tags is included in Additional data file 5.

### RT-PCR

Total RNA from *X. tropicalis *and *X. laevis *embryoswas isolated using Trizol reagent (Invitrogen, Carlsbad, CA, USA). cDNAs were reverse transcribed with MMLV (Promega, Madison, WI, USA) using oligo-dT. RT-PCR analyses were performed in the exponential phase of amplification using primers listed in Supplementary Table 4 in Additional data file 1. Gels in figures are representative of several independent experiments.

### Tag-mapping

The most recently available *X. tropicalis *genome sequence (Assembly 4.1, August 2005) was downloaded from the JGI web site [64]. In addition to the genome sequence, four independent databases with transcripts of *X. tropicalis *were also used, which contain both partial and full-length transcripts. These databases are Ensembl, NCBI, Unigene and Gurdon. The Ensembl database contains a total of 28,657 RNAs, out of which 4,067 cDNAs have a known 3'-UTR sequence. The NCBI database contains 7,976 mRNA sequences. The Unigene database consists of 42,654 EST clusters (unigenes). The Gurdon database contains 41,921 EST clusters generated by the Gurdon Institute [42]. For each transcript in these databases, all potential tags were extracted and sequentially numbered, starting from the 3'-end. The presence of downstream internal polyA stretches was also considered to renumber the tag position within a transcript, since priming of the oligo-dT to these regions is likely to occur, generating truncated cDNAs [43]. The lists with the virtual tags from these databases, along with the calculated information mentioned above, were consolidated into a single table.

Similarly, as previously described [31], a table containing all potential virtual tags extracted from the genome sequence, integrated with the known genome annotation, was generated. This table contains the complete list of genomic tags with their positions in the genome (beginning, end, strand, scaffold), their frequency of occurrence both in the genome and in the cDNA databases, genome annotation (if it maps or not to an annotated element) and detailed transcript mapping information (5'-UTR, coding region, 3'-UTR, upstream and close to an internal polyA stretch, tag position from the 3'-end). In the case of transcripts from Ensembl with unknown 3'-UTR information, a fixed and continuous region in the genome with a length of 1,793 nucleotides downstream of the stop codon was assigned as a predicted 3'-UTR. This length was selected because more than 95% of the known 3'-UTRs of *X. tropicalis *(from Ensembl) are shorter than this and because more than 92% of these 3'-UTRs are contained in a single exon. All these tables (genomic and transcriptomic) were generated by our SAGE tool kit software, which runs on Linux OS and is freely available upon request. The tag-mapping procedure was carried out by simply comparing the experimental tags against the virtual libraries of genomic and transcript databases described above. In contrast to our original methodology of 'hierarchical gene assignment' described for yeast [31], in this work we did not classify the tags into different confidence types because many of the tags have multiple genomic occurrences (only 3.3% of experimental tags match to a single genome position), and thus many tags would end up with an undefined confidence type. In the case where a tag mapped to an intergenic region, it was also recorded if a transcript was annotated in the opposite strand or not. The lists of experimental tags in each category shown in Figure 1 and the tag-mapping of experimental tags in each transcript database are included in Additional data files 6-14.

### Reverse SAGE

Total RNA was extracted from dorsal and ventral explants of *X. tropicalis *gastrula using Trizol (Invitrogen, Carlsbad, CA, USA). This RNA was treated with DNAse I (Invitrogen, Carlsbad, CA, USA) and the rSAGE protocol was performed as previously described [47]. rSAGE products were extracted from the gel, purified and ligated to the pGEM-T vector for sequencing. A product of rSAGE was defined as specific if it fulfilled the following conditions: it must contain the entire SAGE tag; it must contain the primer rSAGE R1; it must contain a polyA tract; and the rSAGE product without the polyA tract must have an exact match (100% sequence identity and no gaps) against the genome. These requirements are necessary to exclude sequences derived from PCR artifacts. rSAGE sequences (tag and downstream sequence) were searched in the genome sequence using BLAT [65] and Ensembl BLAST [66]. Additionally, the rSAGE sequences were also compared with BLAST to the *X. tropicalis *transcripts available at the NCBI database [67].

Additional large data files used in this work containing the complete genome sequence of *X. tropicalis*, all transcript sequence databases and the full genomic library of experimental tag sequences along with the genome annotation can be downloaded directly from our web site [68].

## Abbreviations

BMP: bone morphogenetic protein; EST: expressed sequence tag; NCBI: National Center for Biotechnology Information; rSAGE: reverse SAGE; SAGE: serial analysis of gene expression; UTR: untranslated region.

## Authors' contributions

FF, FM and JL planned and designed the experiments. FF prepared SAGE libraries, tables for tag-mapping, and performed SAGE data analysis, RT-PCR experiments and rSAGE. NS was involved in rSAGE and performed *in situ *hybridizations. FM, JC and IAV developed all the required custom software and databases for bioinformatics analysis. FF, FM and JL wrote the manuscript.

## Additional data files

The following additional data are available with the online version of this paper. Additional data file 1 includes Supplementary Tables 1-4 and their legends. Additional data file 2 is a table listing the 180 tags with *p*-values < 0.05 (obtained with the three statistical tests), information about known genes and count fold-ratios. Additional data file 3 is a figure showing RT-PCR for *DV11 *and *DV17 *in dorsal and ventral explants of *X. tropicalis*. Additional data file 4 contains the complete list of experimental tags with their frequencies of occurrence in the SAGE libraries, normalized count ratio and *p*-values from three different statistical tests. Additional data file 5 lists the 15-nucleotide tag sequences, their frequencies in the SAGE libraries, count ratios, *p*-values and their corresponding sequences of 14-nucleotide tags. Additional data file 6 lists the experimental tags matching to the genome and to the Ensembl transcripts in positions 1, 2 or polyA-next. Additional data file 7 lists the experimental tags matching to the genome, and to the NCBI, Unigenes and Gurdon transcript databases in positions 1, 2 or polyA-next (only those not present in Ensembl). Additional data file 8 lists the experimental tags matching to the genome but without reliable matches to the transcript databases. Additional data file 9 lists the experimental tags matching to the genome but without any match to the transcript databases. Additional data file 10 lists the experimental tags matching to the genome and their frequencies of occurrence in the transcript databases used in this study. Additional data file 11 lists results of tag-mapping of experimental tags to the Ensembl database. Additional data file 12 lists results of tag-mapping of experimental tags to the NCBI database. Additional data file 13 lists results of tag-mapping of experimental tags to the Unigene database. Additional data file 14 lists results of tag-mapping of experimental tags to the Gurdon database.

## Supplementary Material

Additional data file 1 Supplementary Table 1 lists the tags derived from known genes with differential expression along the dorso-ventral axis and their frequency of occurrence in our SAGE libraries. Supplementary Table 2 lists the tag-mapping to transcripts for the 125 tags with significant difference of frequency of occurrence between both libraries. Supplementary Table 3 lists reverse SAGE sequences. Supplementary Table 4 lists the sequences of the primers and information about the probes used in this study. Click here for file

Additional data file 2 The 180 tags with *p*-values < 0.05 (obtained with the three statistical tests), information about known genes and count fold-ratios. Click here for file

Additional data file 3 RT-PCR for *DV11 *and *DV17 *in dorsal and ventral explants of *X. tropicalis*. Click here for file

Additional data file 4 Experimental tags with their frequencies of occurrence in the SAGE libraries, normalized count ratio and *p*-values from three different statistical tests. Click here for file

Additional data file 5 The 15-nucleotide tag sequences, their frequencies in the SAGE libraries, count ratios, *p*-values and their corresponding sequences of 14-nucleotide tags. Click here for file

Additional data file 6 Experimental tags matching to the genome and to the Ensembl transcripts in positions 1, 2 or polyA-next. Click here for file

Additional data file 7 Experimental tags matching to the genome, and to the NCBI, Unigenes and Gurdon transcript databases in positions 1, 2 or polyA-next (only those not present in Ensembl). Click here for file

Additional data file 8 Experimental tags matching to the genome but without reliable matches to the transcript databases. Click here for file

Additional data file 9 Experimental tags matching to the genome but without any match to the transcript databases. Click here for file

Additional data file 10 Experimental tags matching to the genome and their frequencies of occurrence in the transcript databases used in this study. Click here for file

Additional data file 11 Tag-mapping of experimental tags to the Ensembl database. Click here for file

Additional data file 12 Tag-mapping of experimental tags to the NCBI database. Click here for file

Additional data file 13 Tag-mapping of experimental tags to the Unigene database. Click here for file

Additional data file 14 Tag-mapping of experimental tags to the Gurdon database. Click here for file
