# Supplementary material for: Identification of novel transcripts with differential dorso-ventral expression in Xenopus gastrula using serial analysis of gene expression
Source: Genome Biol. 2009 Feb 11;10(2):R15. doi: 10.1186/gb-2009-10-2-r15 (PMC2688288; doi:10.1186/gb-2009-10-2-r15)
Supplement: Additional data file 1 — Supplementary Table 1 lists the tags derived from known genes with differential expression along the dorso-ventral axis and their frequency of occurrence in our SAGE libraries. Supplementary Table 2 lists the tag-mapping to transcripts for the 125 tags with significant difference of frequency of occurrence between both libraries. Supplementary Table 3 lists reverse SAGE sequences. Supplementary Table 4 lists the sequences of the primers and information about the probes used in this study. [file gb-2009-10-2-r15-S1.pdf]

Faunes et al, Supplementary table 1

Gen	Tag (15th base)	Transcript*	Dorsal frequency	Ventral frequency	Normalized ratio (p-val eSAGE)
admp	CATGTACTGCGTTA(T)	NM_001045692.1 ENSXETT00000019176	8	0	8.2 (0.0035)
cerberus	CATGGATGCATCTA(C)	NM_203515.1	3	0	3.1 (0.1215)
*chordin**	CATGATGTTACTTT(G) CATGGTTCACGATG(G)	Xt6.1-TNeu120o03.3**	3 2	0 0	3.1 (0,1215) 2.1 (0,2465)
dkk-1	CATGATCCTCTCTA(T)	Xt6.1-TGas131c10.3 ENSXETT00000014461	1	0	1 (0,4999)
pintallavis	CATGAAACCTGTTT(T)	NM_203607.1 ENSXETT00000055323	34 (32)	2	17.5 (6,65e-9)
crescent (frzb2)	CATGCGTGGGACCC(G)	NM_001016688.1	2	0	2.1 (0,2465)
goosecoid	CATGATGTGGGATC(T)	NM_001016704.2 ENSXETT00000050392	11	0	11.3 (0,0004)
noggin 1	CATGTTATATGCAC(T)	AY672091.1	2	0	2.1 (0,2465)
otx2	CATGGCTGCCTTCT(G)	NM_001016177.2 ENSXETT00000034219	5	0	5.1 (0,02955)
siamois	CATGAAGTTTGAAG(A)	NM_001097352.1	0	0	-
sox3	CATGAAAGTAAAAC(G)	NM_001007501.1	20 (18)	8	2.6 (0,0204)
xnot	CATGATTGTATTAT(T)	CT025280.2 ENSXETT00000025770	3	0	3.1 (0,1215)
zic3	CATGCAACACTGAA(A)	NM_001005691.1 ENSXETT00000052875	5 (4)	0	5.1 (0,0295)
frzb	CATGGACTTTAATA(C)	NM_001005438.1 ENSXETT00000002846	1	0	1 (0,4999)
sfrp2	CATGTTGTGCCGGT(A)	NM_203731.1 ENSXETT00000055200	1	0	1 (0,4999)
follistatin	CATGTAAATGTGTA(A)	NM_001008056.1	1	0	1 (0,4999)
bambi	CATGGTGCCAGTGA(C)	NM_001008193.1 ENSXETT00000009856	0	6	-5.8 (0,0171)
bmp4	CATGGAGACTCTGA(A)	AJ315161.1	0	2	-1.9 (0,2604)
bmp7	CATGATATTTTATC(C)	NM_001033938.2 ENSXETT00000035775	2 (1)	5	-2.5 (0,3075)
crossveinless-2	CATGTGAAAACTGG(A)	NM_001044402.1	0	1	1 (0,5138)
sizzled	CATGCTGTATAAGA(A)	NM_001044506.1 ENSXETT00000048172	0	1	1 (0,5138)
twisted	CATGCTTACACTAA(T)	NM_001006747.1	0	0	-
wnt8	CATGTTTGGTTTGT(T)	NM_001017208.2 ENSXETT00000008323	0	1	1 (0,5138)
vent1.1	CATGGGAGCTATGG(C)	NM_001114235.1	0	15	-14.6 (3,8e-5)
vent1.2	CATGTAACCCCTGG(A)	NM_203530.1 ENSXETT00000019491	0	8	-7.8 (0,0044)
vent2	CATGATTTTATTCA(C)	AY187016.1 ENSXETT00000056265	8	12	-1.5 (0,4190)
xbra	CATGTGATGTTTTT(G)	NM_001008138.1 ENSXETT00000011786	7	3	2.4 (0,2151)
odc1	CATGGCATTCTCCC(T)	NM_001005441.1 ENSXETT00000007603	31	25	1.3 (0,3790)
ef1alpha	CATGAGGCAGACGG(T)	NM_203970.2	133 (130)	144 (137)	-1.1 (-)

Supplementary Table 1. Tags derived from control genes.

Known genes with differential expression along the dorso-ventral axis were analyzed and the most 3’ tag was extracted. These tags were searched in the list of experimental tags and frequencies in each library are indicated. The 15th base of the tag is indicated between parentheses. If the frequency of occurrence of the 15-nts tag is different from the frequency of the 14-nts tag, this is indicated between parentheses.

* Sequences used to extract virtual tags are indicated. Virtual tags derived from mRNA sequences (“NM”) or Ensembl cDNAs (“ENSXETT”) are first considered. If mRNA sequence or Ensembl cDNA is not available, Unigenes and EST clusters are used.

** Virtual tag for *chordin* was obtained by comparison of sequences of *X. tropicalis* and *X. laevis* to determine the 3’ end. In addition, tag derived from upstream an internal polyA tract position was considered.

Faunes et al, Supplementary table 2

DV_ID	Tag sequence	Dorsal frequency	Ventral frequency	Normalized ratio*	Matches to Genome	Tag-mapping
**DV01**	**CATGAAACCTGTTT**	**34**	**2**	**17.5**	**23**	***Pintallavis (forkhead boxa4)***
**DV02**	**CATGAGTTTCTTGT**	**20**	**1**	**20.6**	**14**	***Xt6.1-CAAM15738.5SS, Xt6.1-CAAM9567.5S, Xt6.1-CAAM4052.5***
**DV03**	**CATGGGAGCTATGG**	**0**	**15**	**-14.6**	**10**	***Vent-1.1***
**DV04**	**CATGCATTAATGAA**	**18**	**2**	**9.3**	**26**	**Not assigned**
**DV05**	**CATGAGCTGTATGC**	**20**	**3**	**6.9**	**1482**	***Doublecortin domain containing 2B, Str#S23608264, family with sequence similarity 119, DCN1-like protein 2, Str#S22844133, Str#S38479083***
**DV06**	**CATGATGTGGGATC**	**11**	**0**	**11.3**	**2**	***goosecoid***
**DV07**	**CATGTGGCGTTCCG**	**9**	**0**	**9.3**	**1**	***Zcsl-2***
**DV08**	**CATGGAAATAAAAC**	**9**	**0**	**9.3**	**82**	***Ubadc1/ LOC496648***
**DV09**	**CATGCAAGTATGTT**	**11**	**1**	**11.3**	**20**	***Sox11***
**DV10**	**CATGTACTGCGTTA**	**8**	**0**	**8.2**	**7**	***admp***
**DV11**	**CATGATGGCGGAGC**	**8**	**0**	**8.2**	**3**	***LOC100124861***
**DV12**	**CATGCTCTTACCCT**	**13**	**2**	**6.7**	**9**	***LOC549498***
**DV13**	**CATGCTAGACTTTT**	**1**	**11**	**-10.7**	**12**	***Id-2***
**DV14**	**CATGTGGGAACAGA**	**0**	**8**	**-7.8**	**10**	**Not assigned**
**DV15**	**CATGTAACCCCTGG**	**0**	**8**	**-7.8**	**11**	***Vent-1.2***
**DV16**	**CATGCGCTGTTACA**	**10**	**1**	**10.3**	**3**	***MGC147163***
**DV17**	**CATGATTGGTGCCC**	**12**	**2**	**6.2**	**11**	***Str.45862/Str.40022***
**DV18**	**CATGAAAATTAAAT**	**12**	**2**	**6.2**	**68**	***LOC548724***
**DV19**	**CATGTTCACTGTTT**	**7**	**0**	**7.2**	**26**	**Not assigned**
**DV20**	**CATGTCTACTGCAG**	**7**	**0**	**7.2**	**20**	***LOC394509***
**DV21**	**CATGTCCTTATTAA**	**7**	**0**	**7.2**	**26**	**Not assigned**
**DV22**	**CATGGTTGGCTGGA**	**1**	**10**	**-9.7**	**9**	***smagp***
**DV23**	**CATGTTTCCAGGTG**	**2**	**12**	**-5.8**	**19**	***Xt6.1-CAAL21480.5S, Xt6.1-XZG37850.5S***
**DV24**	**CATGGCAGCTCCAT**	**2**	**12**	**-5.8**	**44**	***e2f5***
**DV25**	**CATGATGTACAGTT**	**2**	**12**	**-5.8**	**27**	***cyclin A2***
**DV26**	**CATGTTTTAGTTTG**	**0**	**7**	**-6.8**	**45**	***LOC549800***
**DV27**	**CATGTGATACTAAG**	**0**	**7**	**-6.8**	**7**	***MGC76280***
**DV28**	**CATGTAGCTTATAC**	**0**	**7**	**-6.8**	**12**	***hsbp1***
**DV29**	**CATGTAAAACTGAA**	**0**	**7**	**-6.8**	**47**	***LOC100038289***
**DV30**	**CATGGTTAGGAGGT**	**0**	**7**	**-6.8**	**7**	**Not assigned**
**DV31**	**CATGGGGTGGGAGG**	**0**	**7**	**-6.8**	**42**	***MGC75957***
**DV32**	**CATGCCAATTTCGC**	**0**	**7**	**-6.8**	**2**	**Not assigned**
**DV33**	**CATGGAGATTAGCG**	**9**	**1**	**9.3**	**4**	***tumor protein p53***
**DV34**	**CATGCCGCTCAGAC**	**9**	**1**	**9.3**	**3**	***symplekin***
**DV35**	**CATGATGGGATTCT**	**11**	**2**	**5.7**	**12**	***LOC100145022***
**DV36**	**CATGACGAGTAGCG**	**11**	**2**	**5.7**	**0**	**No match to genome**

**DV37**	**CATGTGTGAGAGCA**	**1**	**9**	**-8.8**	**17**	***LOC550036, LOC549521***
**DV38**	**CATGTAGATATTAG**	**1**	**9**	**-8.8**	**25**	***nap1l1***
**DV39**	**CATGGGCAATGGGA**	**1**	**9**	**-8.8**	**16**	***mix1***
**DV40**	**CATGGAATTGAATA**	**1**	**9**	**-8.8**	**60**	***xtr-mir-427-3***
**DV41**	**CATGATTACCTTTT**	**1**	**9**	**-8.8**	**29**	***LOC100158535***
**DV42**	**CATGTGTTTTTATA**	**6**	**0**	**6.2**	**93**	***SMAD 9, splicing factor, arginine/serine-rich 10***
**DV43**	**CATGTGGCCAGCGT**	**6**	**0**	**6.2**	**6**	***splicing factor, arginine/serine-rich 6***
**DV44**	**CATGTGGAGATAGA**	**6**	**0**	**6.2**	**5**	***RNA binding motif protein 13***
**DV45**	**CATGGCGGTACCGG**	**6**	**0**	**6.2**	**2**	***Str#S23705173***
**DV46**	**CATGGCACCGGATA**	**6**	**0**	**6.2**	**2**	**Not assigned**
**DV47**	**CATGGAATACTATA**	**6**	**0**	**6.2**	**18**	***cone-rod homeobox (crx)***
**DV48**	**CATGTCCTTCGTTA**	**2**	**11**	**-5.4**	**2**	***calcium activated nucleotidase 1***
**DV49**	**CATGTACCTCCAAT**	**2**	**11**	**-5.4**	**2**	***Xt6.1-XZG44017.5***
**DV50**	**CATGAAAAGGACTG**	**2**	**11**	**-5.4**	**9**	***transmembrane protein 32***
**DV51**	**CATGTTCTAAATGT**	**0**	**6**	**-5.8**	**44**	***Dnajc2-prov protein***
**DV52**	**CATGTTCGTGGAGG**	**0**	**6**	**-5.8**	**1**	***LOC496724***
**DV53**	**CATGTTACAACTTG**	**0**	**6**	**-5.8**	**10**	***Integral membrane protein GPR137B (Transmembrane 7 superfamily member 1 protein)***
**DV54**	**CATGTGAGTTTGTG**	**0**	**6**	**-5.8**	**13**	***phosphatidylinositol transfer protein, alpha (pitpna), Xt6.1-CABH10159.5SS***
**DV55**	**CATGTATGTACACC**	**0**	**6**	**-5.8**	**16**	***Xt6.1-TTbA059c13.3***
**DV56**	**CATGTACTAAAAAT**	**0**	**6**	**-5.8**	**46**	***MGC69221***
**DV57**	**CATGGTGCCAGTGA**	**0**	**6**	**-5.8**	**16**	***Bambi***
**DV58**	**CATGGGTTAAGGTT**	**0**	**6**	**-5.8**	**19**	***nucleobindin 1***
**DV59**	**CATGGCACTTGTTT**	**0**	**6**	**-5.8**	**21**	***fatty acid binding protein 3, muscle and heart***
**DV60**	**CATGGAAGAGGCAA**	**0**	**6**	**-5.8**	**27**	***Str#S23215713, Str#S34494240, Uncharacterized protein KIAA0574 (Fragment)***
**DV61**	**CATGCTCAACCTGG**	**0**	**6**	**-5.8**	**5**	**Not assigned**
**DV62**	**CATGCTAAGTTATT**	**0**	**6**	**-5.8**	**7**	***Str#S22514784,***
**DV63**	**CATGATGTACAATT**	**0**	**6**	**-5.8**	**15**	***ENSXETT00000054938, ADP-ribosylation factor 3***
**DV64**	**CATGGAAATGCAGT**	**8**	**1**	**8.2**	**35**	***Xt6.1-TTbA027n08.3SS***
**DV65**	**CATGACAAACACAA**	**8**	**1**	**8.2**	**58**	***Glutathione peroxidase 7 precursor (EC 1.11.1.9) (CL683), Brd7-prov protein***
**DV66**	**CATGTGAAATAAAT**	**10**	**2**	**5.1**	**93**	***MGC75584, calcitonin gene-related peptide-receptor component protein (rcp9), Xt6.1-CAAN7840.3, Xt6.1-CABA4576.5***
**DV67**	**CATGGCGGAAGTGA**	**10**	**2**	**5.1**	**7**	***Ribonuclease P protein subunit p38***
**DV68**	**CATGACAAGCTAAG**	**10**	**2**	**5.1**	**5**	**Not assigned**
**DV69**	**CATGTACATAAGTT**	**1**	**8**	**-7.8**	**17**	***Death-associated protein-like 1***

**DV70**	**CATGTAAAGTTGAC**	**1**	**8**	**-7.8**	**12**	***60S ribosomal protein L10a (CSA-19), Str#S20516889, Xt6.1-CAAP3220.3***
**DV71**	**CATGGGGAAGTTTG**	**1**	**8**	**-7.8**	**13**	**Not assigned**
**DV72**	**CATGATGTAAATGC**	**1**	**8**	**-7.8**	**22**	***zona pellucida glycoprotein 4***
**DV73**	**CATGAGTACAAAAT**	**1**	**8**	**-7.8**	**24**	***Xt6.1-CABD13995.3, Xt6.1-TNeu063n12.3SS, Xt6.1-XZG53362.5***
**DV74**	**CATGTTTTTGTTTT**	**2**	**10**	**-4.9**	**260**	***MOB1, Mps One Binder kinase activator-like 1B, ENSXETT00000045544, Str#S16960692***
**DV75**	**CATGACATTTTAGG**	**2**	**10**	**-4.9**	**17**	***lamin B1***
**DV76**	**CATGTGTGCAGAGA**	**5**	**0**	**5.1**	**29**	***flap structure specific endonuclease 1***
**DV77**	**CATGTGCCACAGTG**	**5**	**0**	**5.1**	**27**	***Str#S42160483***
**DV78**	**CATGTCTCTATTAA**	**5**	**0**	**5.1**	**21**	**Not assigned**
**DV79**	**CATGTCCCGTACAT**	**5**	**0**	**5.1**	**0**	**No match to genome**
**DV80**	**CATGTCAGAGCATC**	**5**	**0**	**5.1**	**9**	***Xt6.1-TNeu051c17.5***
**DV81**	**CATGGTTTTGGCAC**	**5**	**0**	**5.1**	**10**	***Str#S43286247***
**DV82**	**CATGGTTCACTTGT**	**5**	**0**	**5.1**	**8**	***LOC548815***
**DV83**	**CATGGTGTCTGTGT**	**5**	**0**	**5.1**	**62**	***ADP-ribosylation factor related protein 1***
**DV84**	**CATGGGAAGGCAAA**	**5**	**0**	**5.1**	**40**	***Lysophosphatidic acid receptor 2, Xt6.1-XZF1171.3S***
**DV85**	**CATGGCTGCCTTCT**	**5**	**0**	**5.1**	**18**	***orthodenticle homeobox 2***
**DV86**	**CATGGCAATGATCA**	**5**	**0**	**5.1**	**21**	**Not assigned**
**DV87**	**CATGGATAAGCTAC**	**5**	**0**	**5.1**	**7**	**Not assigned**
**DV88**	**CATGCTTTTATTTT**	**5**	**0**	**5.1**	**103**	***Str#S17733131, Xt6.1-XZT67122.5, Xt6.1-TTbA020f14.5SS***
**DV89**	**CATGCTGGTTGCAA**	**5**	**0**	**5.1**	**13**	***Str#S26249311***
**DV90**	**CATGCTGAAATTAG**	**5**	**0**	**5.1**	**23**	***Regulator of G-protein signaling 7-binding protein (R7 family-binding protein)***
**DV91**	**CATGCCTATCGGCA**	**5**	**0**	**5.1**	**1**	***Xt6.1-TEgg042i09.3***
**DV92**	**CATGCAGAGCAGAA**	**5**	**0**	**5.1**	**29**	***UTP15, U3 small nucleolar ribonucleoprotein, homolog (utp15)***
**DV93**	**CATGCAACACTGAA**	**5**	**0**	**5.1**	**19**	***Zic family member 3 heterotaxy 1***
**DV94**	**CATGATATCGTTTA**	**5**	**0**	**5.1**	**2**	**Not assigned**
**DV95**	**CATGACTGAATAAA**	**5**	**0**	**5.1**	**39**	***Str#S16958610***
**DV96**	**CATGAAAATGTTAA**	**5**	**0**	**5.1**	**59**	***Xt6.1-TGas120k14.3***
**DV97**	**CATGAAAATGCTAA**	**5**	**0**	**5.1**	**55**	***Interferon-related developmental regulator 2 (SKMC15 protein)***
**DV98**	**CATGTTTTACAATA**	**0**	**5**	**-4.9**	**43**	***Xt6.1-CABJ1926.3***
**DV99**	**CATGTTATACAGTT**	**0**	**5**	**-4.9**	**37**	***ENSXETT00000023000* (without description)**
**DV100**	**CATGTTAACCAACA**	**0**	**5**	**-4.9**	**8**	**Not assigned**
**DV101**	**CATGTTAACATTTG**	**0**	**5**	**-4.9**	**34**	**Not assigned**
**DV102**	**CATGTTAAACCTGA**	**0**	**5**	**-4.9**	**12**	**Not assigned**
**DV103**	**CATGTGCCAAAAAT**	**0**	**5**	**-4.9**	**49**	***Str#S21734651***

**DV104**	**CATGTCTTACTTAC**	**0**	**5**	**-4.9**	**7**	***Str#S37580355***
**DV105**	**CATGTCATTTTTGC**	**0**	**5**	**-4.9**	**57**	***Xt6.1-XZT41995.3***
**DV106**	**CATGTAACTACCCC**	**0**	**5**	**-4.9**	**13**	***Nck-associated protein 1 (NAP 1) (p125Nap1) (Membrane-associated protein HEM-2)***
**DV107**	**CATGGGTGCGATGA**	**0**	**5**	**-4.9**	**2**	***PRP31 pre-mRNA processing factor 31 homolog (prpf31)***
**DV108**	**CATGGATTTGCTCA**	**0**	**5**	**-4.9**	**26**	***CDC20 cell division cycle 20 homolog***
**DV109**	**CATGCCCCTTAAGT**	**0**	**5**	**-4.9**	**4**	**Not assigned**
**DV110**	**CATGCCCATATATA**	**0**	**5**	**-4.9**	**41**	***MGC145307***
**DV111**	**CATGCAGAAGGAAT**	**0**	**5**	**-4.9**	**26**	***Xt6.1-XZT56795.5***
**DV112**	**CATGACTCTGTAGC**	**0**	**5**	**-4.9**	**6**	***MAP3K12 binding inhibitory protein 1 (mbip)***
**DV113**	**CATGAAGGAAATGC**	**0**	**5**	**-4.9**	**14**	***LOC549814***
**DV114**	**CATGAAAATGTGGG**	**0**	**5**	**-4.9**	**22**	***protein phosphatase 1, regulatory subunit 15A, Phospholipid hydroperoxide glutathione peroxidase, mitochondrial precursor***
**DV115**	**CATGTTTGATTGGT**	**7**	**1**	**7.2**	**21**	***voltage-dependent anion channel 1 (vdac1)***
**DV116**	**CATGTGTAAAAAGA**	**7**	**1**	**7.2**	**39**	***MGC69468 protein, Xt6.1-CAAO9219.3SSS***
**DV117**	**CATGGTACTGGAAA**	**7**	**1**	**7.2**	**14**	***MGC89748***
**DV118**	**CATGCTTCCCACCA**	**7**	**1**	**7.2**	**10**	***transmembrane and coiled-coil domain family 2 (tmcc2), Str#S17793010***
**DV119**	**CATGTTTCTTCCAG**	**1**	**7**	**-6.8**	**17**	***Str#S22770715***
**DV120**	**CATGTGTTTATATT**	**1**	**7**	**-6.8**	**238**	***Str#S22514775, Xt6.1-CABI14076.3SS, Xt6.1-CABD514.3SS***
**DV121**	**CATGTGTATTTATC**	**1**	**7**	**-6.8**	**26**	***Str#S30067137***
**DV122**	**CATGCAGGAGTTTT**	**1**	**7**	**-6.8**	**21**	***Hairy and enhancer of split 1, (Hairy2)***
**DV123**	**CATGATAAAAAAAA**	**1**	**7**	**-6.8**	**272**	***26 transcripts***
**DV124**	**CATGACTGAGATAA**	**1**	**7**	**-6.8**	**9**	***Str#S35898101***
**DV125**	**CATGAAGGAGGGTG**	**1**	**7**	**-6.8**	**11**	***MGC69188***

Supplementary Table 2. Tag-mapping of tags with significant differential expression between dorsal and ventral libraries.

Tags with statistical significant difference of frequency of occurrence between SAGE libraries (3 tests, $p < 0.05$) and a fold-ratio of 5 or higher are indicated (listed by p-value, not shown). Reliable tag-mappings (position 1, 2 or polyA-next) in the transcript databases are indicated with the description of each gene when it is present (Ensembl or NCBI). For tags without matches in Ensembl cDNAs or NCBI mRNA sequences, Unigenes and/or Gurdon EST clusters are indicated. When several matches are found, all of them are indicated, but if position 1 and position 2 are present, only transcripts with match in position 1 are indicated.

Faunes et al, Supplementary Table 3

DV_tag	rSAGE sequence	Genome position/transcript
DV01.1	CATGAAACCTGTTTTTCCTTATGTCTGTGCTATTC<br>TTGATATTAATATATAAACTTGTTCAATTAAAAA<br>AAAAAAAAAAAAAAAAAAAAAA	scaffold_474:87,681-87,744<br>*pintallavis*
DV01.2	CATGAAACCTGTTTTTCCTTATGTCTGTGCTATTC<br>TTGATATTAATATATAAACTTGTTCAATAAAAAA<br>AAAAAAAAAAAAAAAAAAAAA	scaffold_474:87,681-87,744<br>*pintallavis*
DV01.3	CATGAAACCTGTTTTTCCTTATGTCTGTGCTATTC<br>TTGATATTAATATATAAACTTGTTCAATTAAAAA<br>AAAAAAAAAAAAAAAAAAAAAAA	scaffold_474:87,681-87,744<br>*pintallavis*
DV04.1	CATGCATTAATGAATGTAAAAAAAAAAAAAAAA<br>AAAAAAAAAAAA	scaffold_19023: 2428-2444
DV04.4	CATGCATTAATGAATGAAAAAAAAAAGAAAAAA<br>AAAAAAAAAAAAA	scaffold_19023: 2428-2444
DV04.11	CATGCATTAATGAATGAAAAAAAAAAAAAAAAA<br>AGAAAAAAAA	scaffold_19023: 2428-2444
DV05.1	CATGAGCTGTATGCCACAATCATCAATATTAAAA<br>CAAGAAAAAAAA	178 genomic matches<br>Cluster Str.39849
DV05.2	CATGAGCTGTATGCCACAATCATCAATATTAAAA<br>CAAGAAAAGGCTTGAACTACTAAAAAAAAAAAA<br>AAAAAAAAAAAAAAA	190 genomic matches<br>Cluster Str.39849
DV05.3	ATGAGCTGTATGCCACAATCATCAATATTAAAAC<br>AAGAAAAGGCTTGAACTACTTCAAAAAAAAAAA<br>AAAAAAAAAAAAAAAAA	199 genomic matches<br>Cluster Str.39849
DV05.4	CATGAGCTGTATGCCACAATCATCAATATTAAAA<br>CAAGAAAAAAAAAAAAAAAAAAAAAAAAAAAAA	179 genomic matches<br>Cluster Str.39849
DV06.3	CATGATGTGGGATCTGTACATAAAGTGTAAATAT<br>TATCATTTCATTGCCTGTAAATAAAAAAGAATGT<br>GCAGGCATTTTGCACAGATACACTGTACTGAATT<br>TACTATTTATACCTTAAGTTAAAGGAATTGCTAC<br>AATAAACAGATGGCAAAAAAAAAAAAAAAAAA<br>AAAAGAAAAAAAAA	scaffold_185:1,843,070-1,843,218<br>*goosecoid*
DV07.3	CATGTGGCGTTCCGTAAAAAAAAAAAAAAAAAA	scaffold_321:185,446-185,460<br>(SNP G→A, BASE 14)<br>*zcsl-2*
DV08.3	CATGGAAATAAAACAAAAAAAAAAAAAAAAAA	scaffold_1309:71,568-71,581<br>*ubadc1*<br>scaffold_328: 504,732- 504,745<br>*LOC496648*
DV08.7	CATGGAAATAAAACAAAAAAAAAAAAAAAAAA<br>AAAA	scaffold_1309:71,568-71,581<br>*ubadc1*<br>scaffold_328: 504,732- 504,745<br>*LOC496648*
DV08.8	CATGGAAATAAAACCTCTTTATGCAAAAAAAAA<br>AAAAAAAAAAAAAAAAAAAAA	scaffold_1309:71,558-71,581<br>*ubadc1*
DV09.1	CATGCAAGTATGTTGGTAACGTTATGTCCTGGTT<br>AAAGGATTAAAATAATTAAGAAAAAAAAAAAAA<br>AAAAAAAAAAAAAAAAAAA	scaffold_125:1,689,068-1,689,121<br>*sox11*
DV09.2	CATGCAAGTATGTTGGTAACGTTATGTCCTGGTT<br>AAAGGATTAAAATAATTAAGAAAAAAAAAAAAGA<br>AAAAAAAAAAAAAAAA	scaffold_125:1,689,068-1,689,121<br>*sox11*
DV10.13	CATGTACTGCGTTATTACTCTTTGTAATATTTGTA<br>TATAAGCAAATTGTTAATATATAATGCTTGCATT<br>GTAAATCTGTAAACTTAACGTGTGAGGATAATTT<br>TCTTTTTTATAAATGTACATATTAAAATATATGCA<br>TTAAAATATAATTTCTATGCAAGAACCTTGGAAA<br>AAAAAAAAAAAAAAAAAAAAAAA	scaffold_112:387,252-387,420<br>*admp*
DV12.5	CATGCTCTTACCCTAAGGAAGGGGCTGACTCCTC<br>AGCCCTTCCCTTCAGAGGTTTTAATATGTCTTTCA<br>GATGCAGTTATAAATAAAGAGGTATTTTGTGTAA<br>CTGCCAAAAAAAAACTTTCTGTAATAAAAGCTTA<br>ATTTGAAATGTAAAAAAAAAAAAAAAAAAAAAA<br>AAAAAAAA	scaffold_411:704,144-704,291<br>*LOC549498*

DV12.6	CATGCTCTTACCCTAAGGAAGGGGCTGACTTCTC<br>AGTCCTTCCCTTCAGAGGTTTTAACATGTCTTTCA<br>GATGCAATTATAAATAAAGAGGTATTTTGTGTAA<br>CTGCCAAAAAAAAAACTTTCTGTAATAAAAGCTTA<br>ATTTGAAGTGCAAAAAAAAAAAAAAAAAAAAAAA<br>AAAAA	scaffold_411:704,144-704,291<br>*LOC549498*
DV12.8	CATGCTCTTACCCTAAGGAAGGGGCTGACTTCTC<br>AGTCCTTCCCTTCAGAGGTTTTAATATGTCTTTCA<br>GATGCAGTTATAAATAAAGAGGTATTTTGTGTAA<br>CTGCCAAAAAAAAAACTTTCTGTAATAAAAGCTTA<br>ATTTGAAATGTAAAAAAAAAAAAAAAAAAAAAAA<br>AAAAAAA	scaffold_411:704,144-704,291<br>*LOC549498*
DV12.9	CATGCTCTTACCCTAAGGAAGGGGCTGACTTCTC<br>AGTCCTTCCCTTCAGAGGTTTTAATATGTCTTTCA<br>GATGCAGTTATAAATAAAGAGGTATTTTGTGTAA<br>CTGCCAAAAAAAAAACTTTCTGTAATAAAAGCTTA<br>ATTTGAAAGGTAAAAAAAAAAAAAAAAAAAAAAA<br>AAAAAAAA	scaffold_411:704,144-704,291<br>*LOC549498*
DV13.2	CATGCTAGACTTTTTATAAAAGTTTGGTTGTAAA<br>GCCTTTTTTTATACAGAATAAATCGATCGTGTTTA<br>TGGAAAAAAAAAAAAAAAAAAAAAAAAAAA	scaffold_125:418,146-418,217<br>*id-2*
DV13.3	CATGCTAGACTTTTTATAAAAGTTTGGTTGTAAA<br>GCCTTTTTTTATACAGAATAAATCGATCGTGTTTA<br>TGGAAAAAAAAAAAAAAAAAAAAAAAAAAA	scaffold_125:418,146-418,217<br>*id-2*
DV13.4	CATGCTAGACTTTTTATAAAAGTTTGGTTGTAAA<br>GCNCTTTTTTTATACAGAATAAATCGATCGTGTTT<br>ATGAAAAAAAAAAAAAAAAAAAAAAAAAAAAA	scaffold_125:418,146-418,217<br>*id-2*
DV14.3	CATGTGGGAACAGATGTATATGATGATTCATTAT<br>CTTGACATTCTTGAAATCAGATATTTTTGTCATTC<br>TCCTTCAGTAAGCGATGCCAATGAAAAAGGGAA<br>GCATTATGTTTACAGATGTGATTATTGGAATGTTT<br>GCCAAAATATTTGGTATCTCTAATAAACTTTGCC<br>TCCAGCGTTGAAAAAAAAAAAAAAAAAAAAAAAA<br>AAAAA	scaffold_58:1,610,508-1,610,688<br>Cluster Str.65896
DV16.2	CATGCGCTGTTACAAAAAACCCTACTTGTACAGG<br>AGCTGTTTGTTTCAACAGAAACAGATCAATAAAT<br>GACATCAATATTTAAAAATAAAAGAAAAAAAAA<br>AAAAAAAAAAAAAAAAAAAAAAA	scaffold_353:66,955-67,035<br>*MGC147163*
DV16.5	CATGCGCTGTTACAAAAAACCCTACTTGTACAGG<br>AGCTGTTTGTTTCAACAGAAACAGATCAATAAAT<br>GACATCAATATTAAAAAAAGAAAAAAAAAAAAA<br>AAAAAAA	scaffold_353:66,955-67,035<br>*MGC147163*
DV17.1	CATGATTGGTGCCCTTTAAGGAGCAAAAAAAAA<br>ACAAAA	scaffold_27:677,348-677,371<br>Cluster Str.40022
DV17.5	CATGATTGGTGCCCTTTAAGGAGCAAAAAAAAA<br>ACAAAATAAAAAAAAAAA	scaffold_27:677,348-677,371<br>Cluster Str.40022
DV18.1	CATGAAAATTAAATGCCCGTTTTCCGAAAAAAAA<br>AAAAAAAAAAAAAAAAAAAA	scaffold_69:2,316,073-2,316,097<br>*LOC548724*
DV18.3	CATGAAAATTAAATGCCCGTTTTCCGAAAAAAAA<br>AAAAAAAAAAAAAAAAAAA	scaffold_69:2,316,073-2,316,097<br>*LOC548724*

Supplementary Table 3. Reverse SAGE sequences and transcript assignment.

For each tag, the specific rSAGE sequences of different clones and their genome and transcript matches are indicated.

Faunes et al, Supplementary Table 4

Transcript	Forward	Reverse	Annealing temperature
Xenopus tropicalis			
ef1α	ACTCAATCACCCAGGACAGATTAGTG	GTGGGTAGTCTGAGAAGGTTTCTACA	55
odc1	GCACATGTCAAGCCAGTTCT	TGCGCTCAGTTCTGGTACTT	60
chordin	ACCCAGAGAGCCAGAGACAA	ATCCACAGGGTCCACAGAAG	55
sizzled	CAGAAGGCCCAGTTGAGTTC	TGATGGATGTCACCAGCAAT	55
DV01	CTGGGAGCAGATTTTGCTGT	TCAAGAATAGCACAGACATAAGGAA	59
DV04	CCCGGGTGGAAAATAGGTAA	GCCTCAATCGAATTGGGTAA	55
DV04 probe	TTGTGCTTTCGGAGCTTTTT	GCCTCAATCGAATTGGGTAA	55
DV05	TGGTGTTGGTCCACTGTGTT	TATGGGGTTCAGGTCAGGAG	55
DV05 probe	TTGTGGAGAAGGACCGATTC	ACTTGGTCGGGAATCCTTTT	55
DV07	TGCTTCTGATTTGCTTGCAG	CTTTGAAGAACTGCCCCTTG	59
DV07 probe	ACTTGCCCCTGACGTATGTT	CATCCCTTGCCTAGAAAACAA	55
DV08	AGTGGCTATTGGGTGACAGG	GTGCGGTTTAAGGTTTGGAA	60
DV08 probe	GACGAGGATCGGGTAGATGA	GGGATTTTGGTATCCGACCT	55
DV09	GGAACATTCCTGCTGTCGTT	CCACAAGGCCTTTTAGATGC	55
DV09 probe	ACCATCAAGTCCGAGTACGG	CCCTCATCCATTTCTCTGGA	55
DV11	GGAGAGTGAGGAGCACAAGG	TTTGGACATTTCCTGCAACA	55
DV12	GTGTCTCCGGTGGCATAACT	GAGAAGTCAGCCCCTTCCTT	57
DV12 probe	ACCTGCCCTGTGTGTAGGAC	ACTGAGGGGTTCCATGTTTG	57
DV13	TTAAACCCGAAACGTCGAAC	AAAAAGGCTTTACAACCAAACTT	56
DV13 probe	CTCCAGCACGTCATCGACTA	GTCGAATGCGGATGGTAAGT	56
DV14	CATGTGGGAACAGATGTATA	CAATGCTGGAGGCAAAGTTT	55
DV14 probe	TTAACGCTGGAGGCAAAGTT	AGGCCTAATGCTCAGGTTCA	55
DV16	CACAGGCTGCATGTTCATCT	TCTGTTGAAACAAACAGCTCCT	59
DV16 probe	TTGTAATCGGAGGAGGATCG	GTCTCCTTCTGTCGCCTTTG	55
DV17	CTTCTCTCGTCACCCTCCTG	ATTTTCTCTGGGCTGTGGTG	55
DV18	TTTGAGTGGCGGCCGAGTGC	CGGGCATTTAATTTTCATGTG	56
DV18 probe	ATGGACGGGTTGTAGCAAAG	GGATTCATTGTGTGGCACTG	55
DV22	GGAGGACTCTTTTGCTGTGG	TCTTTCACCCATCTCCTTGC	60
DV22 probe	CCAGAGGAGGCAATAACCAA	ATAAAAGTGCTGGGCAAGGA	55
DV25 probe	ACAGACCCTAAAAGCGAGCA	ACTCTGCCACTTCAGGAGGA	55
DV38	GCATGTTCTTCACTTGCTTCAT	GCACAGCTATTGCAAGCAGA	59
DV38 probe	AATTGGGCACTTCCTACGTG	TTCAATGGGTGTGGTCTGAA	55
Xenopus laevis			
ef1α	CCTGAACCACCCAGGCCAGATTGGTG	GAGGGTAGTCAGAGAAGCTCTCCACG	55
chordin	CCTCCAATCCAAGACTCCAGCAG	GGAGGAGGAGGAGCTTTGGGACAAG	55
DV07	TGTCATGCGACAGGTAGAGC	GGCTCTGCAACAACTTCTCC	55
DV07 probe	NIBB clon Xl304d08ex		
DV08	TGCTATGCTGGATGAAGACG	TTCATCTTTGGGGTCCTCTG	55
DV08 probe	NIBB clon Xl073g09		
DV09	GCGCCAGTATGTACGAGGAT	TAATCGGGGAAGTCGAAGTG	55
DV09 probe	CGAGAAAATCCCCTTCATCA	TAATCGGGGAAGTCGAAGTG	55
DV12	CTGGTATTCACCTGCCCTGT	ATAGTGGCAGCCAAAAATGG	55
DV12 probe	NIBB clon Xl519d09ex		
DV13	AAACCGCATTCGACTTTTTG	TAGCACCATCTCTGCCACTG	55
DV13 probe	TACAGGCGGCTGAATTCTCT	TAGCACCATCTCTGCCACTG	56
DV14	CTTGCATGCCAGAGTTGAAA	CCGAGCTTTAGTGCCAAGAC	55
DV14 probe	NIBB clon Xl170k05		
DV16	TTGGGTGATTGGTAACAGCA	CTCTGAGATGTTCGGCACAA	56
DV16 probe	NIBB clon Xl516o04ex		
DV18	ACGGCAGGAAGAGAAACTCA	GAGCAAGTCCCAGCAACTTC	56
DV18 probe	NIBB clon Xl261d18ex		55
DV22	AAGACCTTGCAGATGGAGGA	CAGAGAGACCACCAGCATGA	55
DV22 probe	TTGCTGCCGTATTCCTAACC	AAAGCAAAGCCCTCTTGACA	55
DV25 probe	GCCAACAAACAGCCAACTTT	AAGGATCGGCATCAATCAAG	55
DV38	AGCCATCCTCACTGCAGACT	ATCCTCAAGGCCACGTACAC	55
DV38 probe	AAAGGAGGATCCCAAAGGAA	ATCCTCAAGGCCACGTACAC	55

Supplementary Table 4. Primers and NIBB clones used in this study.

Primers used are written from 5’ end to 3’ end (left to right). For *X. laevis*, clones from NIBB used for generating probes are indicated.
