# Supplementary material for: Identification of novel transcripts with differential dorso-ventral expression in Xenopus gastrula using serial analysis of gene expression
Source: Genome Biol. 2009 Feb 11;10(2):R15. doi: 10.1186/gb-2009-10-2-r15 (PMC2688288; doi:10.1186/gb-2009-10-2-r15)
Supplement: Additional data file 3 — RT-PCR for DV11 and DV17 in dorsal and ventral explants of X. tropicalis. [file gb-2009-10-2-r15-S3.pdf]

# Faunes et al, Supplementary Figure S1

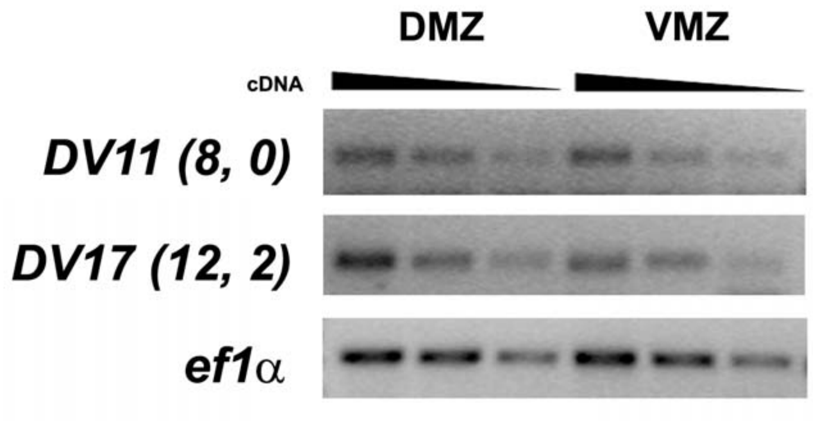


**Supplementary Figure S1.** Total RNA was obtained from dorsal (DMZ) and ventral (VMZ) explants isolated from *X. tropicalis* gastrula stage. Semi-quantitative RT-PCR was performed using specific primers for each transcript. The correct separation of explants was checked using primers for *chordin* and *sizzled* (Figure 3 and data not shown).
